# Disrupted TFEB/GDNF–cAMP/ATP Coupling Underlies Astrocytic Dysfunction and Depression in LRRK2 G2019S Parkinson's Mice

**DOI:** 10.1002/advs.202522066

**Published:** 2026-09-16

**Authors:** Longping Yao, Maryam Hatami, Sumeyye Koc, Qing Jiang, Thomas Skutella, Yuanfeng Zhang

**Affiliations:** ^1^ The Second Affiliated Hospital of Chongqing Medical University Chongqing China; ^2^ Institute for Anatomy and Cell Biology, Heidelberg Medical Faculty Heidelberg University Heidelberg Germany; ^3^ Department of Neuroscience, Institute of Health Sciences Ondokuz Mayıs University Samsun Turkey

**Keywords:** ATP/cAMP, depression, LRRK2 G2019S, PD, TFEB/GDNF

## Abstract

The LRRK2 G2019S mutation, a Parkinson's disease–linked variant, has been associated with depression‐like phenotypes, but mechanisms remain unclear. We chart age‐dependent behavioral changes and astrocyte reactivity in G2019S mice and define a pathway connecting LRRK2 to TFEB/GDNF signaling, cellular energetics, and inflammation. From ∼25 weeks, G2019S carriers show robust depressive‐like behaviors with reduced hippocampal GDNF, exaggerated astrocytic inflammation, diminished ATP, and increased neuronal apoptosis; pharmacologic LRRK2 kinase inhibition reverses these abnormalities. Single‐cell transcriptomics identifies 507 differentially expressed genes enriched for adenylyl cyclase/cAMP signaling, oxidative phosphorylation/ATP metabolism, calcium/ion homeostasis, and cytokine pathways, indicating disrupted cAMP–ATP coupling and perturbed neuroimmune signaling. Mechanistically, LRRK2 G2019S binds and suppresses TFEB while elevating Ser211 phosphorylation, lowering astrocytic GDNF, amplifying inflammation, and reducing ATP. Critically, TFEB overexpression restores GDNF, elevates astrocytic ATP, attenuates inflammatory mediators, and improves depressive‐like behaviors. Enhancing effector nodes is therapeutic: bilateral hippocampal forskolin rapidly (within 6 h) raises cAMP and reverses multiple behavioral measures, and ATP supplementation (systemic or hippocampal) yields comparable benefits. These findings position a LRRK2→TFEB/GDNF→cAMP/ATP axis as a driver of astrocytic inflammation and energetic imbalance underlying depression in G2019S mice and highlight convergent therapeutic strategies targeting LRRK2, TFEB, and ATP/cAMP.

## 1 Introduction

Parkinson's disease (PD) is a progressive neurodegenerative condition that compromises both motor and non‐motor domains [1, 2, 3, 4, 5, 6, 7]. The classical motor syndrome encompasses bradykinesia, resting tremor, rigidity, and postural instability [2, 4, 5, 8]. Yet non‐motor manifestations—cognitive decline, sleep disturbances, autonomic dysfunction, and psychiatric features such as depression—are major determinants of quality of life [2, 9]. Among these, depression is highly prevalent, while its pathophysiological basis remains incompletely defined. PD arises from an interplay between genetic susceptibility and environmental exposures; notably, variants in the leucine‐rich repeat kinase 2 (LRRK2) gene are among the most frequent genetic contributors [10]. Within these, the G2019S substitution is especially common and confers elevated PD risk [2, 6, 11]. This mutation perturbs multiple cellular pathways, promoting neurotoxicity and thereby adding to PD's complex biology [2, 6].

Astrocytes—the most abundant glial population in the central nervous system—sustain neuronal viability, modulate synaptic activity, and supply metabolic support [12]. In neurodegenerative settings, astrocytes frequently enter a reactive state [12, 13]. Current data indicate that astrocytes harboring LRRK2 G2019S acquire heightened neurotoxic properties, which can aggravate neuronal injury and disease progression [6, 14, 15]. However, the precise mechanisms by which G2019S‐mutant astrocytes drive neurotoxicity, particularly within the hippocampus, remain unclear. Transcription factor EB (TFEB) serves as a master regulator of lysosomal biogenesis and autophagy—critical processes for cellular homeostasis and the clearance of damaged organelles and protein aggregates [16]. TFEB dysregulation has been implicated across neurodegenerative disorders, including PD [16], and in astrocytes it is pivotal for neuroprotective responses and the restraint of neuroinflammation [17, 18].

Glial cell line‐derived neurotrophic factor (GDNF) is a potent trophic factor that supports maintenance, regeneration, and survival of dopaminergic neurons, and has therefore been explored as a therapeutic modality in PD [19]. Parallel lines of evidence increasingly connect astrocyte biology to depression [20, 21, 22, 23, 24]. Astrocytes regulate neurotransmitter homeostasis, maintain blood–brain barrier integrity, and shape synaptic transmission and plasticity—processes intimately linked to mood regulation [20, 25]. When astrocytic function is perturbed, imbalances in glutamatergic and GABAergic systems can ensue, contributing to depressive pathophysiology [20, 26]. In PD, astrocyte‐driven neuroinflammation together with impaired trophic support may worsen hippocampal dysfunction, further tying astrocyte activity to depressive symptoms [27].

Here, we investigate how TFEB and GDNF signaling intersect in LRRK2 G2019S–mutant astrocytes and how this crosstalk influences hippocampal neurotoxicity and depressive‐like behavior in PD. We hypothesize that the G2019S mutation disrupts astrocyte function by altering TFEB/GDNF regulation, thereby increasing hippocampal neurotoxicity and fostering depressive phenotypes. By delineating these molecular links, we aim to advance understanding of PD pathophysiology and to identify tractable therapeutic targets for both motor and non‐motor features. Clarifying astrocytic mechanisms in PD may ultimately guide new treatments that slow neurodegeneration and enhance patients’ quality of life.

## 2 Methods

### 2.1 Animals and Management

Transgenic mice expressing LRRK2 (wild type or G2019S) were generated as we previously reported [6], and experimental animals used in the present study were bred and maintained from this existing colony. All procedures were approved by the Ethics Committee of the Second Affiliated Hospital of Chongqing Medical University and were carried out in accordance with the NIH Guide for the Care and Use of Laboratory Animals (National Institutes of Health, Bethesda, MD, USA). Stereotactic intraventricular intubation followed protocols adapted from earlier studies [1, 7]. Mice received bilateral prefrontal cortex (PFC) or hippocampal implants using a stereotactic frame for plasmid delivery (Woruide, Shenzhen, China). Injection coordinates targeting the hippocampus were anteroposterior (AP) −2.3 mm, mediolateral (ML) ±1.8 mm, and dorsoventral (DV) −1.6 mm. For injections targeting the PFC, coordinates were AP +1.8 mm, ML ±0.4 mm, and DV −1.5 mm. To minimize stress‐ and novelty‐related confounds, mice were handled by the experimenter for 3 consecutive days prior to behavioral testing and were habituated to the behavioral testing room for 30 min before each assay. Behavioral tests were performed during the light phase at approximately the same time of day. Apparatuses were cleaned with 70% ethanol between animals to remove olfactory cues if necessary.

For in vivo plasmid delivery, TFEB‐CRISPRa, GDNF‐CRISPRa, TFEB‐CRISPRi, and GDNF‐CRISPRi plasmids (Sangon Biotech, Shanghai, China) were prepared at a final concentration of 1 µg/µL in sterile PBS. NC refers to the negative‐control CRISPRa plasmid carrying the same dCas9‐activator backbone but a non‐targeting (scrambled) sgRNA that does not match any mouse genomic sequence. Plasmids were delivered bilaterally into the hippocampus or PFC using a microsyringe connected to an automated injector mounted on the stereotactic frame. The injection volume was 1 µL per site at a rate of 0.1 µL/min, and the needle was left in place for 5 min after infusion to minimize reflux before slow withdrawal. After surgery, mice were placed on a warming pad until recovery and were monitored daily. Behavioral testing and tissue collection were performed at 3 weeks post‐delivery. Expression and gene modulation efficiency in the injected regions were validated by RT‐qPCR and/or immunoblotting.

An adeno‐associated virus expressing GDNF under the astrocyte‐specific gfaABC1D promoter (AAV‐gfaABC1D‐GDNF) was used to drive astrocyte‐targeted GDNF overexpression in vivo. Mice received a single bilateral stereotaxic injection into the hippocampus (AP −2.3 mm, ML ±1.8 mm, DV −1.6 mm). The viral preparation was delivered at a titer of 1 × 10^1^ ^2^ vg/mL, with an injection volume of 0.5 µL per hemisphere (total 5 × 10^8^ vg per hemisphere) at 0.1 µL/min. The needle was left in place for 5 min after infusion to minimize backflow. Control animals received an equal titer/volume of AAV‐gfaABC1D‐empty. Behavioral testing was performed 3 weeks post‐injection, followed by tissue collection for molecular analyses.

#### 2.1.1 Kinase Inhibitor Administration

MLi‐2 and DNL201 were used to inhibit LRRK2 kinase activity in vivo. Mice received MLi‐2 (10 mg/kg, intraperitoneal injection, twice daily at 12‐h intervals) and DNL201 (10 mg/kg, intraperitoneal injection, twice daily at 12‐h intervals) for 10 consecutive days. Both inhibitors were dissolved in 30% hydroxypropyl‐β‐cyclodextrin (HPβCD; Sigma‐Aldrich, St. Louis, MO, USA) prepared in sterile saline. For behavioral cohorts, behavioral testing was initiated 1 week after the start of drug administration and performed by an experimenter blinded to treatment. For molecular and histological analyses, mice were sacrificed at 1 week after treatment initiation and brain tissues were collected for downstream experiments as described below.

#### 2.1.2 Behavioral Test

##### 2.1.2.1 Sucrose Preference Test (SPT)

The SPT assessed anhedonia—using rodents’ innate preference for sweetness—as a proxy for depressive‐like behavior [28]. Mice underwent a 24‐h acclimation with ad libitum access to 1% sucrose, followed by a 24‐h test. Each animal was provided with two bottles (1% sucrose vs. tap water), and bottle positions were swapped at 12 h to minimize side bias. Prior to testing, mice were water‐deprived for 12 h to standardize thirst. The formal assessment lasted 2 h with concurrent access to both bottles, including a mid‐session side swap. Consumption was determined by weighing bottles before and after. Sucrose preference (%) = [sucrose intake ÷ (sucrose + water intake)] × 100. Mice were habituated to the behavioral testing room for 30 min before the assay.

##### 2.1.2.2 Open Field Test (OFT)

The OFT quantifies exploratory drive and anxiety‐like behavior in a novel arena (100 × 100 cm) enclosed by 50‐cm walls. Sessions last 2 h to capture sustained locomotion and spatial occupancy. Tracking with EthoVision XT 7 (Noldus) yields total distance traveled, center versus periphery dwell time, and ethological measures (e.g., rearing, grooming), providing a quantitative profile of exploration and anxiety. Mice were habituated to the behavioural testing room for 30 min before the session, and the arena was cleaned with 70% ethanol between animals.

##### 2.1.2.3 Forced Swim Test (FST)

Mice were placed in transparent cylinders (height 30 cm; diameter 20 cm) filled with water at 23°C–25°C for a 6‐min trial, with continuous video recording. The primary endpoint was immobility, defined as the absence of active escape‐directed behaviors (e.g., swimming, climbing, or vigorous struggling), with only small movements of the forelimbs and/or hindlimbs necessary to keep the head above water. Each mouse received a 2‐min habituation, after which the final 4 min were scored to quantify immobility as an index of depressive‐like behaviour. To minimize potential novelty‐related artifacts, the first 2 min were considered an adaptation period, and immobility was quantified during the final 4 min.

##### 2.1.2.4 Tail Suspension Test (TST)

Behavioral despair was assessed in a clean white enclosure (55 cm height; 10 cm × 10 cm footprint). Mice were suspended by the tail for a total of 6 min, and behavior was video‐recorded using the SMART tracking system (Version 3.0, Panlab, USA). Immobility—defined as the absence of limb and body movements—served as the primary endpoint. To minimize stress‐related artifacts, a 2‐min acclimatization immediately followed suspension, and only the final 4 min were analyzed.

##### 2.1.2.5 Social Interaction Test (SIT)

Testing took place in a white Plexiglas square arena (30 cm per side; wall height 35 cm), with overhead acquisition at 50 frames/s and analysis in EthoVision XT 11. Each mouse completed two consecutive 10‐min exploration sessions within the same apparatus: first with an empty perforated Plexiglas cage positioned against one wall, and then—with no changes to the arena geometry—after introducing an unfamiliar C57BL/6 male into the perforated cage as the social stimulus. All test mice were male, and stranger mice were also male (sex‐matched). Stranger mice were habituated to the containment cage for 5 min prior to testing. Mice were habituated to the behavioral testing room for 30 min before the session. Social interaction was quantified automatically in EthoVision XT 11 as zone‐based social investigation, defined as the time spent in a predefined interaction zone (5‐cm perimeter) surrounding the containment cage, together with entries into the interaction zone. The arena and cage were cleaned with 70% ethanol between animals to minimize olfactory cues.

##### 2.1.2.6 Light–Dark Test (LDT)

Mice were placed in the dark compartment, and after a 3‐s delay, the entryway to the illuminated chamber opened to permit free exploration for 10 min. Time spent in the light compartment and the number of transitions between compartments were recorded. Following each trial, both chambers were cleaned with alcohol to remove residual odors and standardize conditions. Mice were habituated to the behavioral testing room for 30 min before the assay.

##### 2.1.2.7 ATP Detection in Brain Tissues

Brain tissues from transgenic LRRK2 G2019S and wild‐type (WT) male mice were collected according to established procedures [29]. Mice were euthanized by rapid cervical dislocation, the brains were removed, and placed in ice‐cold artificial cerebrospinal fluid (aCSF; 126 mm NaCl, 26 mm NaHCO_3_, 3 mm KCl, 1.2 mm NaH_2_PO_4_, 2 mm CaCl_2_, 1 mm MgSO_4_, 10 mm glucose). Hippocampal and PFC samples from both hemispheres were then dissected and transferred to 500 µL aCSF for 30 min at room temperature on a gentle rotator. Following centrifugation at 2000 g for 3 min at 4°C, supernatants were harvested for ATP assays. ATP readouts were normalized to each sample's total protein content.

##### 2.1.2.8 ATP Injection

For systemic delivery, mice received intraperitoneal (i.p.) ATP at 125 mg/kg/day or volume‐matched saline once daily for 7 days, followed by behavioral testing. This dosing paradigm was adapted from prior work using systemic ATP supplementation to counteract depression‐related phenotypes in the setting of impaired astrocytic ATP release [30]. Given the physicochemical constraints for blood–brain barrier penetration (e.g., charge and molecular weight) and the rapid extracellular degradation of ATP by ectonucleotidases to adenosine, systemic ATP administration was expected to act predominantly through peripheral and/or metabolite‐mediated purinergic signaling rather than direct ATP entry into the brain [31, 32]. Therefore, to directly test central ATP efficacy, we additionally performed stereotaxic bilateral hippocampal ATP infusion as described below.

For central delivery, animals were positioned in a stereotaxic frame for bilateral hippocampal injections. An automated injector (Pump 11 Elite; Harvard Apparatus, Holliston, MA, USA) administered ATP (25 µm, 1 µL at 0.2 µL/min) or saline at AP = −2.3 mm, ML = ±1.8 mm, DV = −1.6 mm. Behavioral assessments were initiated 4 h after infusion to capture acute effects.

##### 2.1.2.9 Cell Culture

Primary hippocampal astrocytes and neurons were prepared from WT and LRRK2 G2019S mice as indicated. Hippocampi were dissected in ice‐cold HBSS (Gibco, Thermo Fisher Scientific, Waltham, MA, USA), meninges were carefully removed, and tissues were enzymatically dissociated using a Papain Dissociation System (Worthington Biochemical, Lakewood, NJ, USA), followed by gentle trituration. Cells were filtered through a 70‐µm cell strainer (Corning, Corning, NY, USA) and counted prior to plating.

For primary astrocyte culture, dissociated hippocampal cells were plated onto poly‐*D*‐lysine–coated cultureware (Sigma‐Aldrich/Merck, St. Louis, MO, USA) and maintained in DMEM/F12 (Gibco, Thermo Fisher Scientific) supplemented with 10% fetal bovine serum (Gibco, Thermo Fisher Scientific) and 1% penicillin/streptomycin (Gibco, Thermo Fisher Scientific) at 37°C with 5% CO_2_. Astrocytes were enriched using standard purification procedures to reduce non‐astrocytic contamination, re‐plated for experiments, and used at matched culture age across genotypes. Where indicated, astrocytes were treated with LRRK2 kinase inhibitors MLi‐2 or DNL201 (or matched vehicle) for the durations specified in the corresponding experiments prior to downstream assays.

Primary hippocampal neurons were plated on poly‐*D‐*lysine–coated plates or coverslips (Sigma‐Aldrich/Merck) and maintained in Neurobasal medium (Gibco, Thermo Fisher Scientific) supplemented with B27 (Gibco, Thermo Fisher Scientific), GlutaMAX (Gibco, Thermo Fisher Scientific), and 1% penicillin/streptomycin (Gibco, Thermo Fisher Scientific) at 37°C with 5% CO_2_. Half‐medium changes were performed every 3–4 days.

##### 2.1.2.10 ATP Measurement for Cells

ATP was quantified using the ATP assay kit (Promega, Madison, WI) following previously reported protocols [29]. Cultured cells were rinsed three times with pre‐warmed Hanks' Balanced Salt Solution (HBSS; 20 mm Hepes, 0.5 mm MgCl_2_, 0.4 mm MgSO_4_, 1.4 mm CaCl_2_, 5 mm glucose) and then incubated in HBSS at 37°C for 30 min. Supernatants were collected for extracellular ATP determination. For intracellular ATP, HBSS was removed and cells were processed strictly according to the kit manual. Luminescence was recorded on a Spark Control Magellan reader (Tecan, Männedorf, Switzerland). All ATP measurements were normalized to total protein content.

##### 2.1.2.11 Western Blot

Western blotting was carried out as previously described [1, 7]. Total protein was extracted with RIPA lysis buffer (Beyotime, Jiangsu, China) supplemented with protease and phosphatase inhibitors (BioTools, Olathe, KS, USA), following the manufacturers’ instructions. Protein concentrations were determined using the BCA assay (Bio‐Rad Laboratories, Inc., Berkeley, CA, USA). Equal amounts of protein were resolved by SDS‐PAGE (Beyotime Biotechnology, Shanghai, China) and transferred onto PVDF membranes (Millipore, Bedford, USA). Membranes were blocked with 5% TBST and incubated with primary antibodies overnight at 4°C. After three TBST washes, membranes were incubated for 1 h at room temperature with HRP‐conjugated secondary antibodies (appropriate anti‐rabbit IgG; Thermo Fisher Scientific). Antibodies used were: mouse anti‐GDNF (Thermo Fisher Scientific); rabbit anti‐IgG (Thermo Fisher Scientific); rabbit anti‐TFEB (Cell Signaling Technology, Danvers, MA, USA); rabbit anti‐phospho‐TFEB (Ser122) (Cell Signaling Technology, Danvers, MA, USA); rabbit anti‐phospho‐TFEB (Ser211) (Thermo Fisher Scientific); rabbit anti‐phospho‐TFEB (Ser142) (Merck Millipore).

##### 2.1.2.12 Reverse Transcription‐Quantitative Real‐Time Polymerase Chain Reaction (RT‐qPCR)

Total RNA from cell cultures and mouse brain tissues was isolated using RNAiso Plus (Takara, Japan). cDNA was synthesized with the Reverse Transcription Kit (RR047A; Takara, Dalian, China) using random primers (Sangon Biotech, Shanghai, China). qPCR was performed to quantify mRNA levels, with GAPDH as the normalization control for both human and mouse samples. Relative expression was calculated by the ΔΔ*Ct* method. Primer sequences are provided in Table 1.

**TABLE 1:** Primer sequences for RT‐qPCR.

Genes	Primer sequences, 5′–3′ forward	Primer sequences, 5′–3′ reverse
GDNF	TGAAGAGGCTCTCGGAGTCA	GCTGCCGCTTGTTTATCTGG
TFEB	GAAGAACAGGGGTGAGGCAG	CCACTTGTGGGGGAGTTCAG
TNF‐α	TATGGCTCAGGGTCCAACTC	GGAAAGCCCATTTGAGTCCT
IL‐1β	CTCACAAGCAGAGCACAAGC	CAGTCCAGCCCATACTTTAGG
IL‐6	TTCCATCCAGTTGCCTTCTT	CATTTCCACGATTTCCCAGA
CCL2	CCACTCACCTGCTGCTACTC	AAGGCATCACAGTCCGAGTC
CCL5	TGCTCCAATCTTGCAGTCGT	GCAAGCAATGACAGGGAAGC
iNOS	GCTTGGGTCTTGTTCACTCC	TCCTCTTTCAGGTCACTTTGG
CXCL2	GGCGGTCAAAAAGTTTGCCT	TTCTTCCGTTGAGGGACAGC
IL‐17	TCATCCCTCAAAGCTCAGCG	TTCATTGCGGTGGAGAGTCC
GAPDH	GGGAAATTCAACGGCACAGT	AGATGGTGATGGGCTTCCC

##### 2.1.2.13 Transfection

CRISPR guide RNAs (sgRNAs) were designed using CHOPCHOP (https://chopchop.cbu.uib.no/) to target promoter‐proximal regions of the indicated genes for transcriptional modulation. For CRISPR activation (CRISPRa), sgRNAs were selected within the upstream promoter region near the transcription start site (TSS) to maximize transcriptional activation; for CRISPR interference (CRISPRi), sgRNAs were positioned around the TSS to achieve effective repression. sgRNA oligonucleotides were synthesized by Shanghai Shenggong Trade Co., Ltd. (Shanghai, China), cloned into the corresponding sgRNA expression backbone, and all constructs were sequence‐verified prior to use. Cells were transfected with the indicated CRISPRa or CRISPRi plasmids using Lipofectamine 3000 (Invitrogen) according to the manufacturer's protocol. After transfection, cells were maintained under standard culture conditions and harvested at the time points specified in each experiment for downstream analyses. Transfection efficiency was evaluated by RT‐qPCR or western blot.

##### 2.1.2.14 Astrocyte–Neuron Co‐Culture

Astrocyte–neuron crosstalk was assessed using an astrocyte‐conditioned medium (ACM) paradigm. Primary hippocampal astrocytes were prepared from WT or LRRK2 G2019S mice and cultured as described above. For ACM collection, astrocytes were washed once with pre‐warmed medium and then incubated in fresh culture medium. The supernatant was collected after 24 h, centrifuged to remove cellular debris, and immediately applied to WT primary neuronal cultures. WT neurons were exposed to ACM derived from WT or G2019S astrocytes for 48 h, after which neuronal viability, activity, and apoptosis were quantified.

For inhibitor‐rescue experiments, G2019S astrocytes were pretreated with the LRRK2 kinase inhibitors MLi‐2 and DNL201 for 24 h prior to ACM collection. Following pretreatment, astrocytes were washed once and switched to fresh medium to generate ACM. The resulting ACM was applied to WT neurons for 48 h before downstream analyses.

##### 2.1.2.15 Cell Viability Assay

For the CCK8 assay, cells were seeded at 3000 cells/well in 96‐well plates with 100 µL complete medium and kept at 37°C. The next day, 10 µL CCK8 reagent (Beyotime, Shanghai, China) was added per well, and incubation continued for 2 h at 37°C. Plates were then gently shaken for 1 min on an orbital shaker to ensure uniform color development. OD450 was read on a Multiskan microplate reader (Thermo Fisher Scientific, Waltham, MA, USA). Background was corrected using reagent‐only (medium + CCK‐8) wells, and data were expressed relative to the corresponding control condition. Each condition was run in triplicate, and the experiment was repeated three times.

##### 2.1.2.16 Lactate Dehydrogenase (LDH) Assay

LDH release, used as a cytotoxicity readout, was quantified as reported [6]. Cells were plated at 5 × 10^4^ cells/well in 96‐well plates and maintained for 72 h. Following the indicated treatments, culture supernatants were collected and processed with the LDH Cytotoxicity Assay Kit (Thermo Fisher Scientific, Waltham, MA, USA) according to the manual. Absorbance was measured on a Thermo Fisher microplate reader at 490 nm, with 680 nm used as a reference wavelength, and values were background‐corrected by subtracting the reference signal. Relative cytotoxicity was derived by comparing treated versus control groups, followed by statistical analysis of group differences. Where indicated, results were normalized to the control condition and expressed as relative LDH release.

##### 2.1.2.17 Molecular Docking

Protein–protein docking was carried out on HDOCK (http://h‐dockphys.hust.edu.cn/), sampling poses and residue contacts within 5 Å. The TFEB model was taken from the Alphafold database (non‐redundant entries used), and LRRK2 was retrieved from the PDB (PDBID: 7li4). PyMOL (v2.3.0; https://pymol.org) was employed to remove original ligands/organic molecules, dehydrate, and separate protein components. Structures were then hydrogenated/protonated using the “prepare” module in Discovery Studio. Ligplus was used to generate 2D interaction maps, while the “analysis interface” in Discovery Studio characterized the protein–protein interface. Final visualization of interacting amino‐acid residues was produced in PyMOL.

##### 2.1.2.18 Immunoprecipitation Assay

Immunoprecipitation (IP) was conducted following the previously published workflow [6]. Cells were lysed in IP buffer composed of 1% Triton X‐100, 150 mm NaCl, 10 mm NaH_2_PO_4_, 15 mm Na_2_HPO_4_, 50 mm NaF, 1 mm EDTA, and 1 mm Na_3_VO_4_. Cell lysates were first incubated with the specified primary antibodies, followed by incubation with Protein G agarose beads (20 µL per reaction, Millipore, Billerica, MA, USA). Beads were washed with immunoprecipitation buffer, then boiled in 2× sample buffer. The precipitated proteins were subsequently analyzed by western blot. Antibodies used in this assay were rabbit anti‐TFEB (Cell Signaling Technology, Danvers, MA, USA) and rabbit anti‐LRRK2 (Cell Signaling Technology, Danvers, MA, USA).

##### 2.1.2.19 Immunofluorescence Staining

Immunofluorescence staining followed our previous procedure [6]. Animals were anesthetized and underwent transcardial perfusion with PBS, followed by ice‐cold 4% paraformaldehyde. Brains were postfixed in the same fixative for 2 days at 4°C, then cryoprotected in 30% sucrose for an additional 2 days at 4°C. Brain coronal sections (12 µm) were prepared on a freezing microtome from Leica and mounted onto poly‐*d*‐lysine–coated slides. Sections were incubated with the primary antibodies listed below and then with secondary antibodies. Antibodies included: mouse Anti‐GFAP antibody (1:400, Cell Signaling Technology, Danvers, MA, USA); rat Anti‐Neun antibody (1:50, Abcam); rabbit anti‐TFEB antibody (1:50, Thermo Fisher Scientific); rabbit anti‐GDNF antibody (1:50, Thermo Fisher Scientific). Immunoreactivity was visualized as green fluorescence on an LSM 880 laser scanning confocal microscope (Carl Zeiss, Oberkochen, Germany). Images were captured and analyzed using ZEN light software (Carl Zeiss) to enable precise measurement of labeled structures’ fluorescence intensities and spatial resolution.

##### 2.1.2.20 Chromatin Immunoprecipitation–qPCR (ChIP–qPCR)

ChIP–qPCR was performed to assess TFEB occupancy at the GDNF promoter in primary hippocampal astrocytes. Cells were cross‐linked with 1% formaldehyde for 10 min at room temperature and quenched with 0.125 m glycine, followed by two washes with ice‐cold PBS. Chromatin was extracted in ChIP lysis buffer supplemented with protease inhibitors and sheared by sonication to an average size of 200–500 bp. Sheared chromatin was incubated overnight at 4°C with anti‐TFEB antibody or matched IgG control, and immune complexes were captured using Protein A/G beads. Beads were sequentially washed with low‐salt, high‐salt, and LiCl buffers, and cross‐links were reversed at 65°C overnight. ChIP DNA was purified and analyzed by qPCR using SYBR Green chemistry. Enrichment at the GDNF promoter and a distal negative‐control region was calculated after normalization to input DNA and IgG control (ΔΔ*Ct* method), and results are presented as % input and/or fold enrichment as indicated.

##### 2.1.2.21 High‐Performance Liquid Chromatography (HPLC)

HPLC analyses were conducted to quantify aspartate, glutamate, serine, glutamine, glycine, and GABA. All standards and reagents were obtained from Sigma‐Aldrich, except where noted. HPLC‐grade water and acetonitrile were sourced from Thermo Fisher Scientific. The instrumentation comprised an Agilent 1200 series system equipped with a G1354A quaternary pump, 1322A micro vacuum degasser, G1316A column thermostat, G1329A autosampler, a Hipersil ODS column (5‐µm particle size, 4 × 125 mm), and a G1321A fluorescence detector. Sample derivatization utilized o‐phthalaldehyde (OPA) and 9‐fluorenylmethyloxycarbonyl chloride (FMOC). For each sample, 2 µL of borate buffer (0.1 m, pH 10.5) and 2 µL OPA (0.08 m) were mixed with 1 µL of sample; after 60 s, 1 µL FMOC (0.01 m) was added; following another 60 s, 1 µL of the mixture was injected. Mobile phase A was 10 nm phosphate‐buffered saline (PBS) with 0.5% tetrahydrofuran (pH 7.2), and mobile phase B was PBS: methanol: acetonitrile (50:35:15, v/v). The gradient started at 100% A and reached 100% B over 25 min at 1 mL/min, with the column held at 40°C. The fluorescence detection limit was 1 pmol per injection. Amino acids were quantified from peak area ratios against calibration curves, and results were normalized to total protein content in brain slices (BCA protein assay kit, Thermo Fisher Scientific).

##### 2.1.2.22 Statistical Analysis

Data are reported as mean ± standard deviation (SD) from three independent experiments. For comparisons between two groups, a two‐tailed Student's *t‐*test was applied; datasets with more than two groups were evaluated by analysis of variance (ANOVA). A threshold of *p* < 0.05 was used to define statistical significance.

## 3 Results

### 3.1 Age‐Dependent Depression‐Like Behaviors and Neurobiological Alterations in LRRK2 G2019S Mice

**FIGURE 1:**
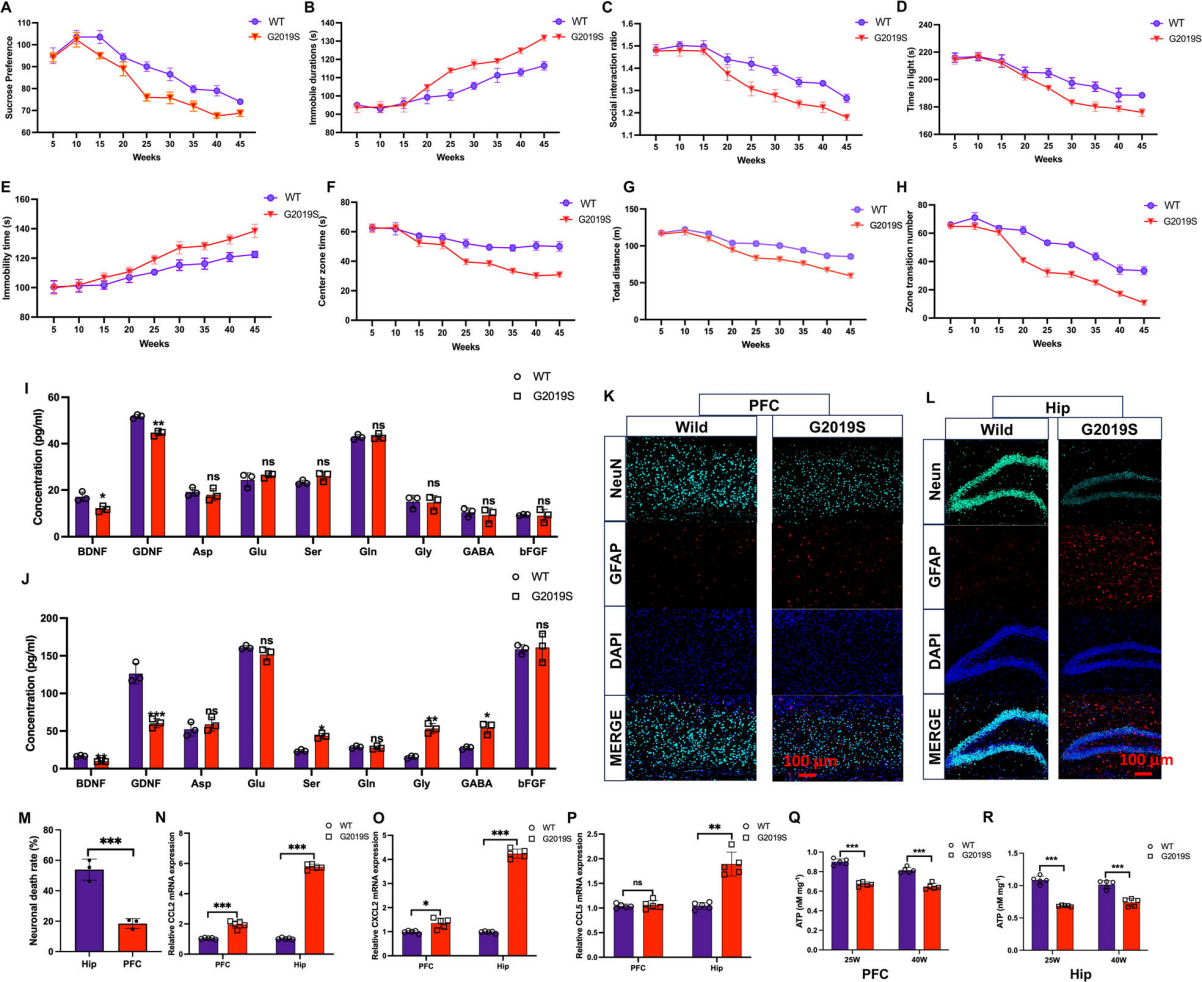
Age‐dependent depression‐like behaviors and neurobiological alterations in LRRK2 G2019S mice. (A–H) Longitudinal behavioral battery in WT and LRRK2 G2019S mice at 5, 10, 15, 20, 25, 30, 35, 40, and 45 weeks: SPT (A), FST (B), SIT (C), LDT (D), TST (E), and OFT readouts (F–H). Longitudinal data were analyzed by two‐way repeated‐measures ANOVA (genotype × time), followed by Sidak's multiple‐comparisons test for WT versus G2019S at each time point. For panels (A–G), significant genotype differences emerged from 25 weeks onward (Sidak‐adjusted *p* < 0.05). For panel H, significant genotype differences emerged from 20 weeks onward (Sidak‐adjusted *p* < 0.05). (I,J) Quantification of BDNF, GDNF, Glu, Asp, Ser, Gln, Gly, and GABA in slice media from PFC (I) and hippocampus (J). (K,L) Confocal immunofluorescence showing NeuN and GFAP in PFC (K) and hippocampus (L). Color code: green = anti‐NeuN, red = anti‐GFAP. Scale bar = 50 µm. (M) NeuN mean fluorescence intensity (background‐subtracted) was quantified within ROIs in the hippocampal dentate gyrus (Hip) and PFC and normalized to the WT group. (N–P) RT‐qPCR of inflammatory transcripts normalized to GAPDH: CCL2 (N), CXCL2 (O), and CCL5 (P). (Q,R) ATP measurements in PFC (Q) and hippocampal (R) slices. Data are mean ± SD. Replicates: behavioral tests *n* = 12, RT‐qPCR *n* = 5, ATP slices *n* = 5, others *n* = 3. Statistics: * *p* < 0.05, ** *p* < 0.01, *** *p* < 0.001, ns = not significant.

Prior work suggested that LRRK2 G2019S can precipitate depressive phenotypes in mice [33], yet mechanistic detail remains sparse. To systematically profile this phenotype, we tracked WT and LRRK2 G2019S cohorts across 9 time points—5, 10, 15, 20, 25, 30, 35, 40, and 45 weeks. No group differences were detected at 5–15 weeks. Beginning at 20 weeks, however, G2019S mice showed a clear depressive‐like signature: greater immobility in FST and TST, lower sucrose preference in SPT, reduced social interaction, and altered exploration/anxiety indices in OFT and LDT (Figure 1A–H).

Based on the longitudinal behavioral profiling, we defined 25 weeks as the earliest age at which depression‐like behaviors consistently emerged, and therefore used this time point for subsequent mechanistic analyses unless otherwise stated. Neurotrophic and neurotransmitter profiling in PFC and hippocampus revealed reduced GDNF and BDNF, most prominently in the hippocampus, accompanied by shifts in transmitter content—including increased serotonin (Ser), glycine (Gly), and GABA (Figure 1I,J). Converging histological analyses showed astrocyte activation and neuronal loss, again preferentially in the hippocampus (Figure 1K,L; Figure S16A–C), with neuronal death rates exceeding those in PFC (Figure 1M). Consistent with these changes, inflammatory mediators were elevated; in both regions—but to a greater extent in hippocampus—we detected increases in C–C motif chemokine ligand 2 (CCL2), C–X–C motif chemokine ligand 8 (CXCL2), C–C motif chemokine ligand 5 (CCL5), interleukin 1 beta (IL‐1β), tumor necrosis factor‐alpha (TNF‐α), inducible nitric oxide synthase (iNOS) and interleukin 6 (IL‐6) (Figure 1N–P; Figure S16D–G). Analysis of the GEO dataset GSE265833 further supported tight neuron–astrocyte coupling in the G2019S context, offering a plausible cellular substrate for the observed astrocytosis and neuronal death (Figure S1A,B). These data point to a hippocampus‐biased inflammatory milieu that likely contributes to the behavioral phenotype.

Given that ATP acts as a neurotransmitter/neuromodulator in brain circuits [30] and that astrocyte‐derived ATP dysregulation has been implicated in depression [20], we next tracked ATP across ages. ATP levels were diminished in both PFC and hippocampus, with the hippocampus showing the sharper decline in 25‐ and 40‐week G2019S mice (Figure 1Q,R). This energy imbalance aligns with the emergence and progression of depressive‐like behaviors [20, 34]. Collectively, our longitudinal analysis delineates an age‐linked trajectory in G2019S mice—minimal differences early, followed by behavioral impairment from 20 weeks onward—paralleled by hippocampus‐centric deficits in neurotrophic support, neurotransmitter perturbations, gliosis/neuronal loss, heightened inflammation, and ATP reduction, which together likely drive the depressive‐like state.

### 3.2 GDNF Alleviates Depression‐Like Behaviors and Astrocyte Inflammatory Responses in LRRK2 G2019S Mice

**FIGURE 2:**
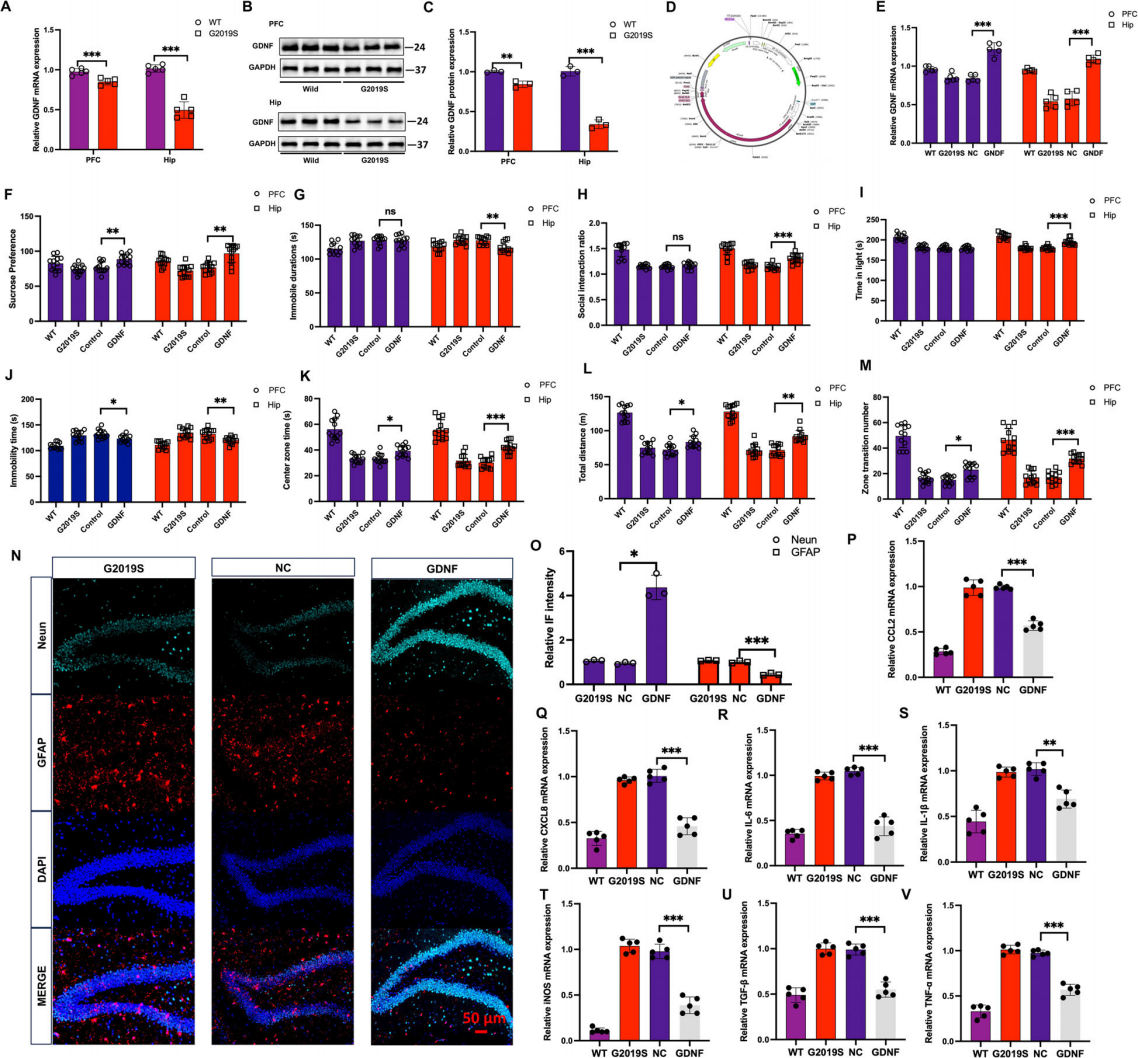
GDNF alleviates depression‐like behaviors and astrocyte inflammatory responses in LRRK2 G2019S mice. (A) RT‐qPCR quantification of GDNF mRNA in PFC and hippocampus from WT and LRRK2 G2019S mice. (B,C) Western blot of GDNF in PFC and hippocampus (B) with relative protein levels quantified (C). (D,E) Schematic of stereotaxic plasmid delivery and validation of CRISPRa‐driven GDNF upregulation in PFC and hippocampus. For CRISPRa experiments, G2019S mice received either a negative‐control CRISPRa vector (Control/NC) or a GDNF‐targeting CRISPRa vector (GDNF). The Control (also referred to as NC) group represents LRRK2 G2019S mice injected with a non‐targeting scrambled sgRNA CRISPRa plasmid, whereas the GDNF group represents LRRK2 G2019S mice injected with the GDNF‐targeting CRISPRa plasmid. WT mice were included as a physiological reference group. NC denotes the negative‐control CRISPRa plasmid containing the same dCas9‐activator backbone with a non‐targeting (scrambled) sgRNA. (F–M) Post‐GDNF CRISPRi injections, depression‐related assays were performed: SPT (F), FST (G), SIT (H), LDT (I), TST (J), and OFT readouts (K–M). (N) Representative heat maps depicting time allocation within the open‐field arena. (O,P) Confocal images of NeuN and GFAP in the hippocampus (O) with relative fluorescence intensities quantified (P). NeuN was quantified as mean fluorescence intensity within the ROI after background subtraction and normalized to the control group; thus, changes reflect NeuN immunoreactivity rather than NeuN+ cell counts or neurogenesis. Color code: green = anti‐Neun, red = anti‐GFAP. Scale bar = 50 µm. (Q–W) RT‐qPCR for inflammatory transcripts normalized to GAPDH: CCL2 (Q), CXCL2 (R), IL‐1β (S), IL‐6 (T), iNOS (U), CCL5 (V), TNF‐α (W). Data presentation: mean ± SD. Replicates: behavioral tests *n* = 12; RT‐qPCR *n* = 5; others *n *= 3. Statistics: one‐way ANOVA with Tukey's multiple comparisons. Significance: * *p* < 0.05, ** *p* < 0.01, *** *p* < 0.001, ns = not significant.

GDNF, a neurotrophic ligand within the TGF‐β superfamily, is essential for the survival, maturation, and function of multiple neuronal classes in the CNS [35] and is best known for supporting dopaminergic neurons vulnerable in PD [36]. Owing to these neuroprotective actions, GDNF has long been explored as a candidate therapy for neurodegeneration and neural injury [37, 38]. In our experiments, GDNF expression was markedly reduced in both the PFC and hippocampus of LRRK2 G2019S mice, with the largest decrement in the hippocampus (Figure 2A–C). We hypothesized that this deficit contributes to astrocyte activation and neuronal loss. Consistent with this, both mRNA and protein assays confirmed diminished GDNF—again most striking in the hippocampus. To test therapeutic potential, we used CRISPRa to drive GDNF overexpression via stereotaxic injection into PFC and hippocampus (Figure 2D,E). Hippocampal GDNF augmentation robustly improved depression‐like phenotypes across multiple behavioral paradigms (Figure 2F–N). PFC overexpression also produced benefits, but effects were weaker than hippocampal targeting (Figure 2F–N), underscoring the hippocampus as a principal locus for the depressive phenotype in G2019S mice.

Beyond behavior, GDNF overexpression curtailed astrocyte activation and attenuated neuronal injury, as indicated by reduced GFAP reactivity and preserved NeuN immunoreactivity within the hippocampus (Figure 2O–P). In parallel, it suppressed inflammatory mediators—including CCL2, CXCL2, TGF‐β, TNF‐α, IL‐1β, IL‐6, and iNOS (Figure 2Q–W). Taken together, these data indicate that restoring GDNF counters astrocytic activation, neuroinflammation, and neuronal vulnerability, thereby alleviating depression‐like behaviors in LRRK2 G2019S mice.

### 3.3 Inhibition of the Kinase of LRRK2 Improves Depressive Behaviors

**FIGURE 3:**
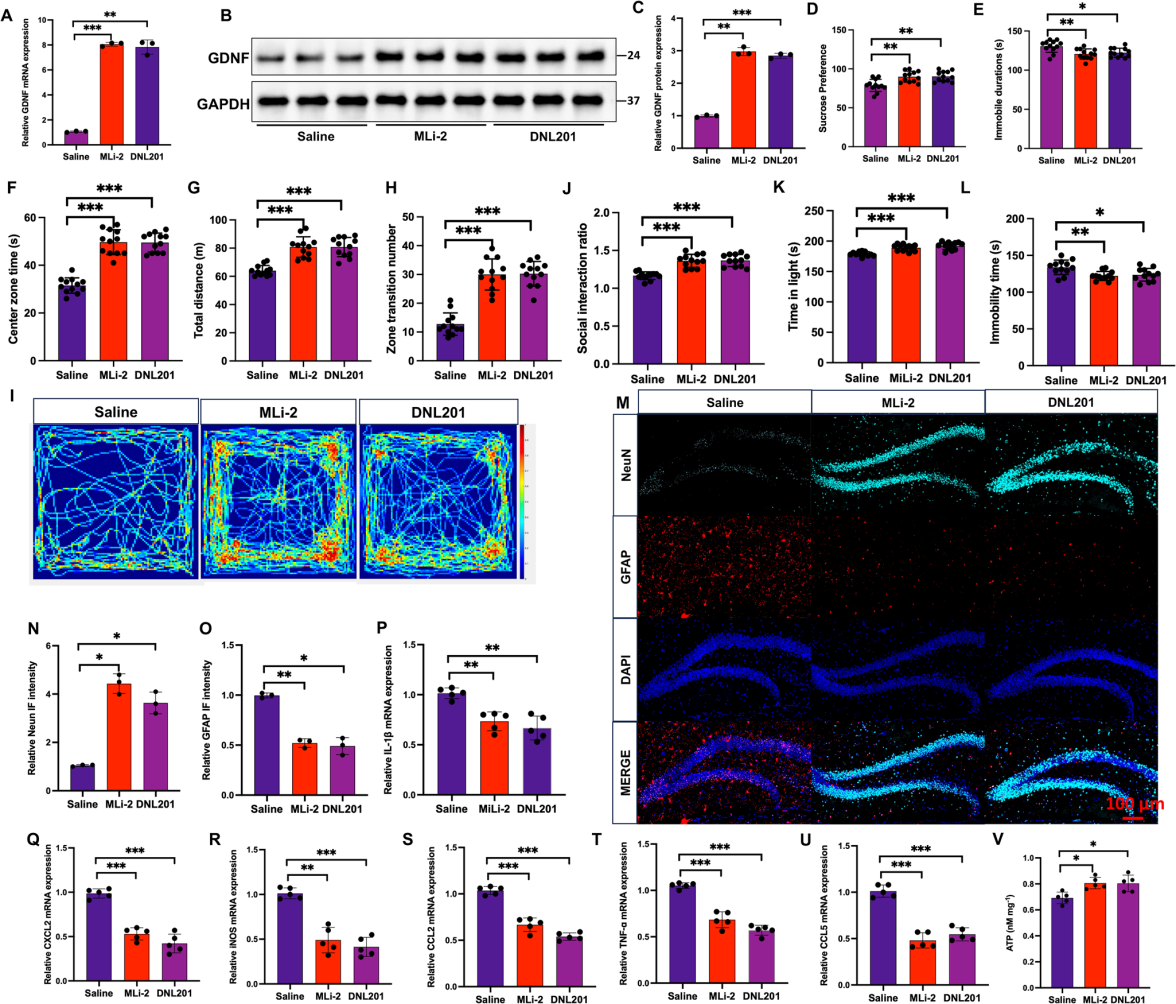
Inhibition of the kinase of LRRK2 improves depressive behaviors. LRRK2 G2019S mice were treated with the kinase inhibitors MLi‐2 and DNL201 to test kinase‐dependent modulation of depressive phenotypes. (A) RT‐qPCR of GDNF mRNA in the hippocampus. (B,C) Western blot for GDNF (B) with quantification (C). (D–L) Post‐GDNF CRISPRi injection, depression‐related assays were performed: SPT (D), FST (E), OFT (F–I), SIT (J), LDT (K), TST (L). (I) shows representative heat maps of time allocation within the open‐field arena. (M–O) Confocal images of Neun and GFAP in the hippocampus after MLi‐2/DNL201 treatment (M), with fluorescence intensity quantified for Neun (N) and GFAP (O). Color code: green = anti‐Neun, red = anti‐GFAP. Scale bar = 50 µm. (P–U) RT‐qPCR of inflammatory transcripts (normalized to GAPDH): IL‐1β (P), CXCL2 (Q), iNOS (R), CCL2 (S), TNF‐α (T), CCL5 (U). (W–X) ATP levels measured in medium from hippocampal slices. Data: mean ± SD. Replicates: behavioral tests *n* = 12; RT‐qPCR *n* = 5; ATP slices *n* = 5; others *n* = 3. Statistics: one‐way ANOVA with Tukey's multiple comparisons. Significance: * *p* < 0.05; ** *p* < 0.01; *** *p* < 0.001.

Given that the LRRK2 G2019S variant elevates kinase activity [6], we posited this enzymatic gain might underlie the depressive phenotypes in mutant mice. To interrogate this, MLi‐2 and DNL201—two selective, potent LRRK2 kinase inhibitors—were administered to LRRK2 G2019S cohorts. Both compounds increased GDNF expression in the hippocampus (Figure 3A–C). Concordantly, behavioral batteries showed reversal of depression‐like phenotypes across SPT, OFT, FST, SIT, LDT, and TST (Figure 3D–L).

At the cellular level, LRRK2 kinase blockade via MLi‐2 or DNL201 suppressed astrocyte activation and rescued neuronal death triggered by G2019S (Figure 3M–O). Inflammatory profiling revealed broad downregulation of neurotoxic mediators, including CXCL2, TGF‐β, TNF‐α, IL‐1β, CCL2, IL‐6, and iNOS (Figure 3P–U). Notably, inhibitor treatment also enhanced hippocampal ATP synthesis (Figure 3V), indicating an improvement in energy metabolism that likely supports restoration of neuronal function and resilience (Figure 3V). Collectively, these findings show that pharmacologic inhibition of LRRK2 kinase—by boosting hippocampal GDNF, dampening astrocytic inflammation, and preventing neuronal loss—ameliorates depression‐like behaviors in LRRK2 G2019S mice, underscoring the therapeutic promise of LRRK2 kinase inhibitors in PD.

### 3.4 GDNF Regulates G2019S–Mutant Astrocyte Inflammatory Responses and ATP Release

**FIGURE 4:**
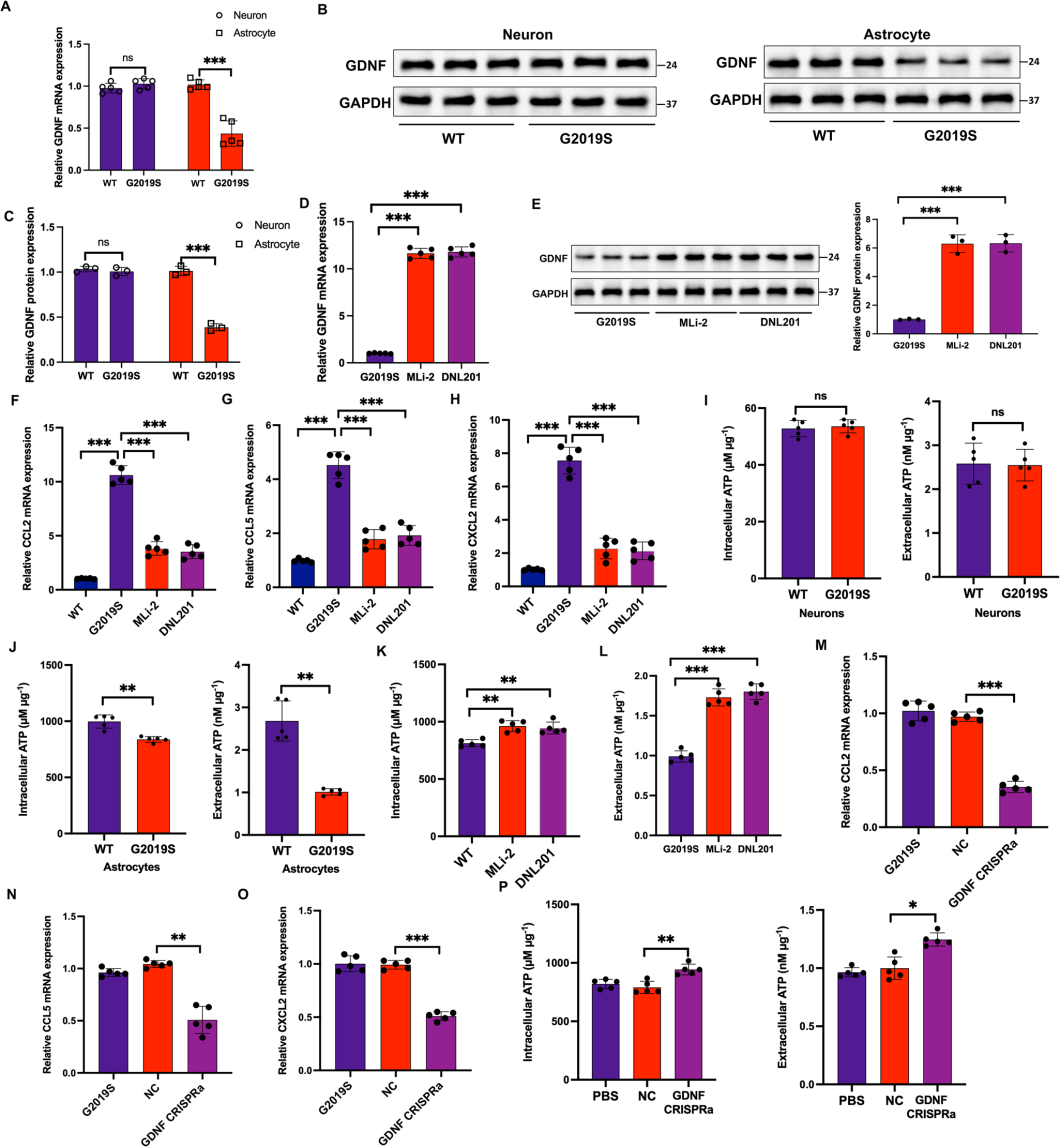
GDNF regulates G2019S–mutant astrocyte inflammatory responses and ATP release. Primary neurons and astrocytes were isolated from the hippocampus of WT and LRRK2 G2019S mice. (A) RT‐qPCR quantification of GDNF mRNA in primary neurons and astrocytes. (B,C) Western blot for GDNF (B) with relative protein levels quantified (C). G2019S astrocyte pharmacology (MLi‐2/DNL201): (D) RT‐qPCR of GDNF mRNA after MLi‐2 and DNL201 treatment. (E) Western blot for GDNF with relative expression calculated. Astrocyte inflammatory readouts: (F–M) RT‐qPCR for CCL2 (F), CCL5 (G), CXCL2 (H), normalized to GAPDH. ATP measurements (genotype effects): (I,J) Intracellular and extracellular ATP in primary cultured neurons (I) and astrocytes (J) from WT and G2019S mice. ATP measurements (kinase inhibition): (K,L) Intracellular (K) and extracellular (L) ATP in G2019S astrocytes after MLi‐2/DNL201 treatment. GDNF gain‐of‐function in astrocytes: Astrocytes were transfected with GDNF CRISPRa plasmids using Lipofectamine 3000. (M–O) RT‐qPCR for CCL2 (M), CCL5 (N), CXCL2 (O), normalized to GAPDH. (P) Intracellular and extracellular ATP in astrocytes after GDNF CRISPRa transfection. Data presentation: mean ± SD. Replicates: ATP measurement *n* = 5; RT‐qPCR *n* = 5; others *n *= 3. Statistics: one‐way ANOVA with Tukey's multiple comparisons. Significance: * *p* < 0.05; ** *p* < 0.01; *** *p* < 0.001; ns = not significant.

To pinpoint the cellular source of GDNF loss in LRRK2 G2019S mice, we profiled primary neurons and astrocytes from the hippocampus. The mutation did not change neuronal GDNF but produced a robust reduction in astrocytic GDNF at both mRNA and protein levels (Figure 4A–C), indicating that the whole‐tissue deficit largely originates from astrocytes rather than neurons. Consistently, treating astrocytes with the LRRK2 kinase inhibitors MLi‐2 and DNL201 restored GDNF expression suppressed by G2019S (Figure 4D,E). Because these inhibitors attenuate hippocampal neuroinflammation in vivo, we next asked whether they directly modulate astrocyte inflammatory tone in vitro. Indeed, both MLi‐2 and DNL201 markedly lowered inflammatory factor expression in astrocytes (Figure 4F–H; Figure S17A–D). We then dissected the origin of ATP dysregulation. In neurons, the mutation left ATP unchanged—for both intracellular production and extracellular release (Figure 4I). In contrast, astrocytes from G2019S mice showed significant decreases in intracellular and extracellular ATP (Figure 4J), establishing an astrocyte‐specific vulnerability that accounts for the hippocampal ATP abnormalities. Importantly, MLi‐2 and DNL201 treatment improved astrocytic ATP in both compartments (Figure 4K,L).

To evaluate direct neuronal consequences of the mutation, we quantified apoptosis in primary neurons from G2019S versus WT mice and observed substantially higher apoptosis in G2019S neurons (Figure S2A,B). We also tested astrocyte‐to‐neuron crosstalk using conditioned media: WT neurons exposed for 48 h to medium from G2019S astrocytes exhibited reduced viability and increased apoptosis (Figure S2C,D). When G2019S astrocytes were pretreated for 24 h with MLi‐2 or DNL201, their conditioned medium rescued neuronal viability and diminished apoptosis after 48 h of exposure (Figure S2E,F), indicating that secreted factors from mutant astrocytes drive neuronal injury and are drug‐modifiable.

Finally, to directly counter astrocytic GDNF loss, we activated GDNF in astrocytes using a CRISPRa plasmid. Western blot and PCR verified successful activation (Figure S3A–C). Mirroring LRRK2 kinase inhibition, GDNF activation in astrocytes strongly suppressed inflammatory factors (Figure 4M–O; Figure S17E–H) and substantially elevated both intracellular and extracellular ATP (Figure 4P). In neuron–astrocyte co‐culture, astrocytic GDNF activation improved neuronal viability and reduced cytotoxicity (Figure S3D,E). Together, these data identify astrocytes as the principal locus for GDNF deficiency and ATP imbalance in the G2019S setting and show that targeting astrocytic dysfunction—via LRRK2 kinase inhibition or GDNF activation—can normalize inflammatory signaling, restore ATP homeostasis, and protect neurons.

### 3.5 LRRK2 Phosphorylates TFEB at Ser211

**FIGURE 5:**
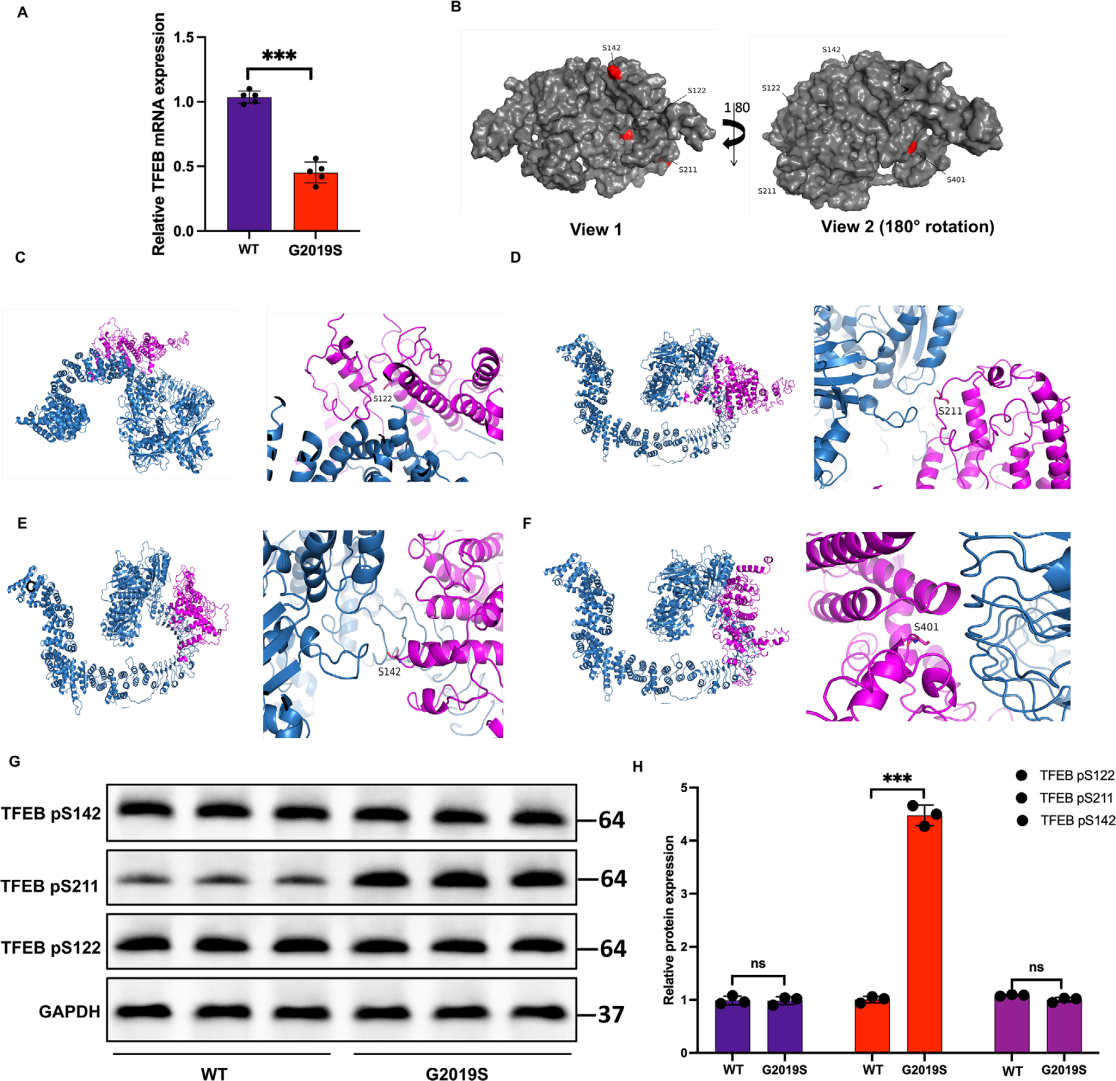
LRRK2 phosphorylates TFEB at Ser211. (A) RT‐qPCR quantification of TFEB mRNA in hippocampal primary astrocytes from WT and LRRK2 G2019S mice. (B) Surface representation of a 3D structural model of TFEB showing candidate phosphorylation residues assessed in this study (S122, S142, S211, and S401; highlighted in red). The same TFEB model is shown in two orientations (View 1 and View 2), with View 2 rotated by 180° relative to View 1. (C–F) Predicted LRRK2–TFEB heteromeric complex illustrating a putative kinase–substrate interface; full‐length LRRK2 rendered in blue, and the TFEB kinase‐target region in purple. (G,H) Immunoblot detection of TFEB phosphorylation using site‐specific antibodies recognizing Ser122, Ser142, and Ser211, showing phosphorylation changes induced by the LRRK2 G2019S mutation. Data are mean ± SD; experiments were repeated by 5 times for RT‐qPCR and 3 times for western blot. Statistical significance: ** *p* < 0.01; ns, not significant.

Prior studies reported that LRRK2 negatively regulates members of the MiT–TFE family, including transcription factor binding to IGHM enhancer 3 (TFE3) and TFEB [39]; however, whether the kinase‐activating G2019S variant perturbs TFEB signaling in astrocytes, and how this may affect neurotrophic support and cellular energetic homeostasis, has remained unclear. In our model, GO/KEGG enrichment implicated cAMP signaling (Figure 7A,B) and in vivo data showed cytokine/chemokine inflammatory responses, two processes in which TFEB has been increasingly implicated as a regulatory node [40, 41]. We therefore prioritized TFEB for mechanistic interrogation and found that LRRK2 G2019S markedly reduces TFEB mRNA (Figure 5A). Because G2019S elevates LRRK2 kinase activity, we further posited that TFEB phosphorylation—a modification that alters TFEB function and localization, thereby limiting target‐gene transcription [42]—would also be affected. Using commercially available phospho‐site–specific antibodies, we assessed TFEB phosphorylation in our previously established induced pluripotent stem cell (iPSC)–induced neuronal cells [6], a system with robust LRRK2 and TFEB expression suitable for initial phospho‐site screening with available antibodies. To guide epitope selection, we built a 3D structural model predicting candidate LRRK2 target residues on TFEB (Figure 5B), which highlighted Ser121, Ser142, Ser211, and Ser401 as plausible sites (Figure 5C–F). We then probed TFEB with antibodies recognizing phospho‐S122, phospho‐S142, and phospho‐S211. Relative to wild type, the G2019S mutation selectively increased TFEB phosphorylation at Ser211, with no comparable elevation at the other tested sites (Figure 5G,H).

### 3.6 LRRK2 G2019S Reshapes Astrocytic ATP and Inflammatory Programs Through TFEB/GDNF–Dependent Interactions

**FIGURE 6:**
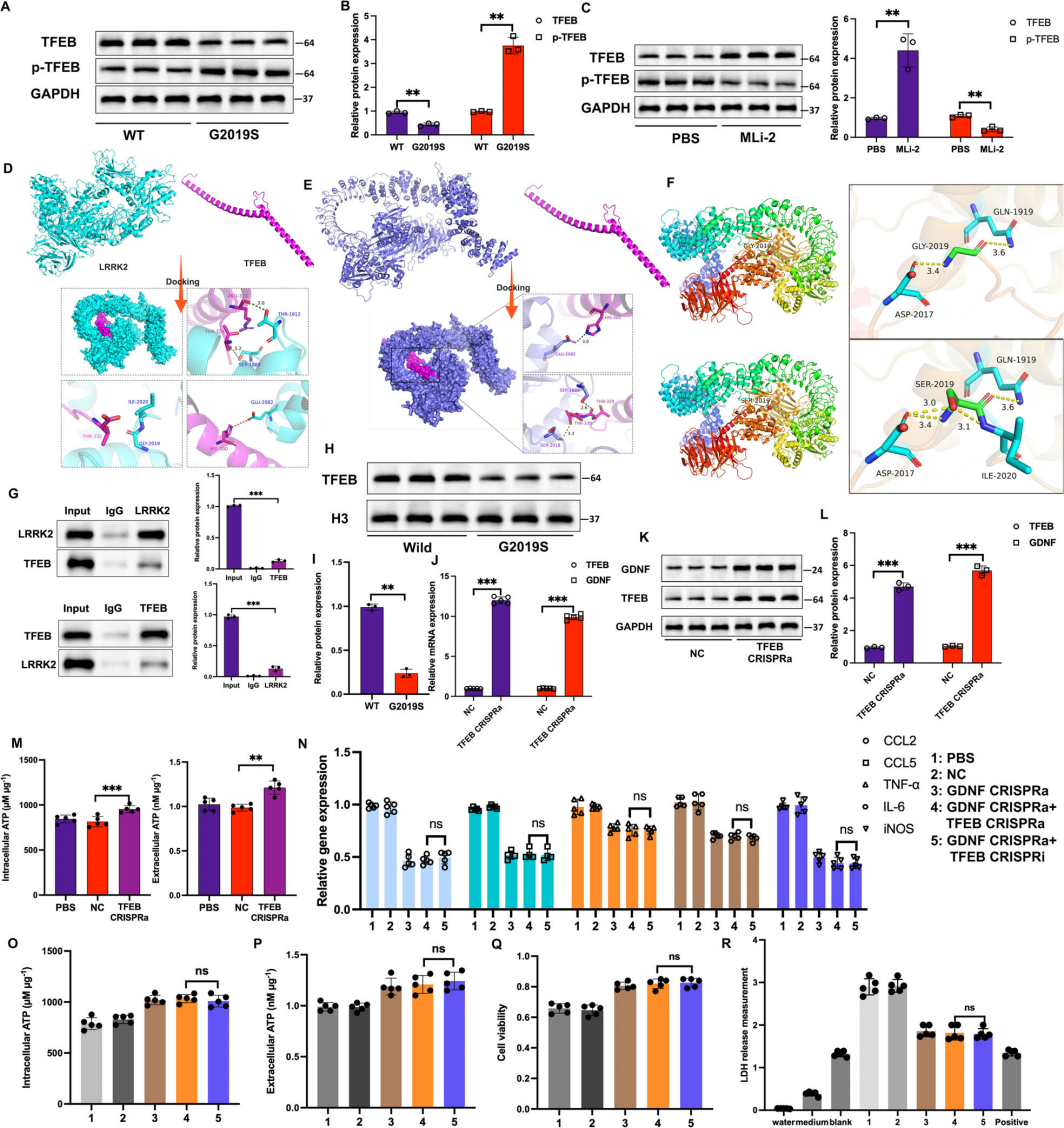
LRRK2 G2019S reshapes astrocytic ATP and inflammatory programs through TFEB/GDNF–dependent interactions. (A,B) Western blot quantification of TFEB and p‐TFEB with relative protein levels calculated. (C) Following MLi‐2 treatment of primary astrocytes, TFEB and p‐TFEB were assessed by Western blot and quantified. (D) Molecular docking supports LRRK2–TFEB binding. Left panel: LRRK2 (sky blue) and TFEB (dark red). Sky‐blue dashed lines = hydrogen bonds; red dashed lines = salt bridges. Notable contacts: LRRK2 GLU2082–TFEB HIS300 (salt bridge); LRRK2 SER1889/THR1912–TFEB ARG323/THR329 (H‐bonds at 3 and 3.2 Å). Hydrophobic interactions involve GLY2019 and ILE330 of LRRK2 with THR330 of TFEB, stabilizing the complex. (E) Right panel: LRRK2 G2019S chain (purple) and TFEB chain (green). Blue/green dashed lines denote hydrogen bonds between TFEB HIS300/THR329/THR330 and LRRK2 GLU2082/SER1889/SER2019 at 3, 2.6, and 3.3 Å, respectively, indicating stable binding. (F) Structural comparison of wild‐type and G2019S LRRK2 kinase domains. (G) Immunoprecipitation and reverse‐immunoprecipitation from primary astrocyte lysates verify LRRK2–TFEB interactions, followed by western blot with LRRK2‐ and TFEB‐specific antibodies. (H,I) After nucleocytoplasmic separation, nuclear TFEB was detected by western blot and normalized to H3. TFEB gain‐of‐function in astrocytes: Primary astrocytes were transfected with TFEB CRISPRa using Lipofectamine 3000. (J) RT‐qPCR confirmation of TFEB and GDNF mRNA. (K,L) Western blot of TFEB and GDNF (K) with relative protein levels (L). (M) Intracellular and extracellular ATP in astrocytes. GDNF loss‐ and TFEB modulation: Astrocytes were first transfected with GDNF CRISPRi for 48 h, then with TFEB CRISPRa or CRISPRi for an additional 48 h. (N) RT‐qPCR readouts for CCL2, CCL5, TGF‐β1, IL‐6, iNOS. (O,P) Intracellular (O) and extracellular (P) ATP measurements in astrocytes. (Q,R) In neuron–astrocyte co‐culture, neuronal apoptosis was quantified by CCK8 (Q) and LDH assays (R). Data presentation: mean ± SD. Replicates: western blot *n* = 3; others *n* = 5. Statistics: one‐way ANOVA with Tukey's multiple comparisons. Significance: ** *p* < 0.01; *** *p* < 0.001; ns = not significant.

Our data indicate that the LRRK2 G2019S mutation reduces TFEB protein abundance—a core MiT–TFE transcription factor—and elevates TFEB phosphorylation in vivo, consistent with dampened transcriptional output (Figure 6A,B). Pharmacological inhibition with MLi‐2 restored TFEB expression while lowering its phosphorylation, pointing to recovery of TFEB activity suppressed by G2019S (Figure 6C; Figure S4A). To define the physical interface between LRRK2 and TFEB, we performed molecular docking as a structural biology/computer‐aided design approach [6]. Docking predicted a binding energy of −130.70 kcal/mol for the LRRK2–TFEB complex (Figure 6D; Figure S5), indicative of a stable interaction. The G2019S variant yielded an even lower energy (−164.28 kcal/mol), implying enhanced complex stability relative to wild type (Figure 6E; Figure S6). Mechanistically, replacing GLY2019 with SER2019 rewires the local hydrogen‐bond network: in wild type, GLY2019 interacts with ASP2017 and GLN1919 at 3.4 and 3.6 Å; after mutation, SER2019 forms new bonds with ILE2020, GLN1919, and ASP2017 at 3.6, 3.1, and 3/3.4 Å, and additionally establishes a hydrogen bond with THR330 on TFEB—an interaction absent for GLY2019 (Figure 6F). Immunoprecipitation confirmed a direct LRRK2–TFEB association (Figure 6G). Functionally, nuclear TFEB content was reduced in G2019S, aligning with limited gene transcription (Figure 6H,I).

We next activated TFEB in astrocytes using CRISPRa plasmids, with PCR and western blot verification (Figure 6N–P; Figure S4B). Given the co‐occurring GDNF deficit in G2019S, we tested whether TFEB governs GDNF expression. TFEB activation increased GDNF (Figure 6J–L), suppressed inflammatory factors in astrocytes (Figure S18A–G), and raised both intracellular and extracellular ATP in hippocampal astrocytes (Figure 6M). To strengthen the causal link between TFEB and GDNF transcription, we performed ChIP–qPCR in primary hippocampal astrocytes. TFEB ChIP showed robust enrichment at the GDNF promoter region containing predicted TFEB‐responsive elements, whereas the distal negative‐control region and IgG control displayed only background‐level signal (Figure S14A). Neuron–astrocyte co‐culture assay showing that astrocytic TFEB activation increases neuronal viability and reduces cytotoxicity (LDH release, D) compared with G2019S and empty‐vector controls (Figure S4C,D). These effects parallel those from GDNF activation, suggesting TFEB acts upstream of GDNF to coordinate astrocytic inflammation and ATP release.

To test GDNF dependence explicitly, we first suppressed GDNF in astrocytes using CRISPR interference (CRISPRi) for 48 h (Figure S4E), then altered TFEB with CRISPRa or CRISPRi for another 48 h. When GDNF was overexpressed, subsequent changes in TFEB did not significantly modify CCL2, CCL5, TGF‐β1, IL‐6, or iNOS (Figure 6N), ATP remained stable (Figure 6O,P), and neuronal apoptosis in co‐culture showed no further change (Figure 6Q,R). Thus, while TFEB can regulate astrocytic inflammatory responses and ATP production, its impact—along with downstream neuronal protection—is largely mediated through GDNF.

### 3.7 Disrupted cAMP–ATP Coupling in LRRK2 G2019S and its Rescue via the TFEB/GDNF

**FIGURE 7:**
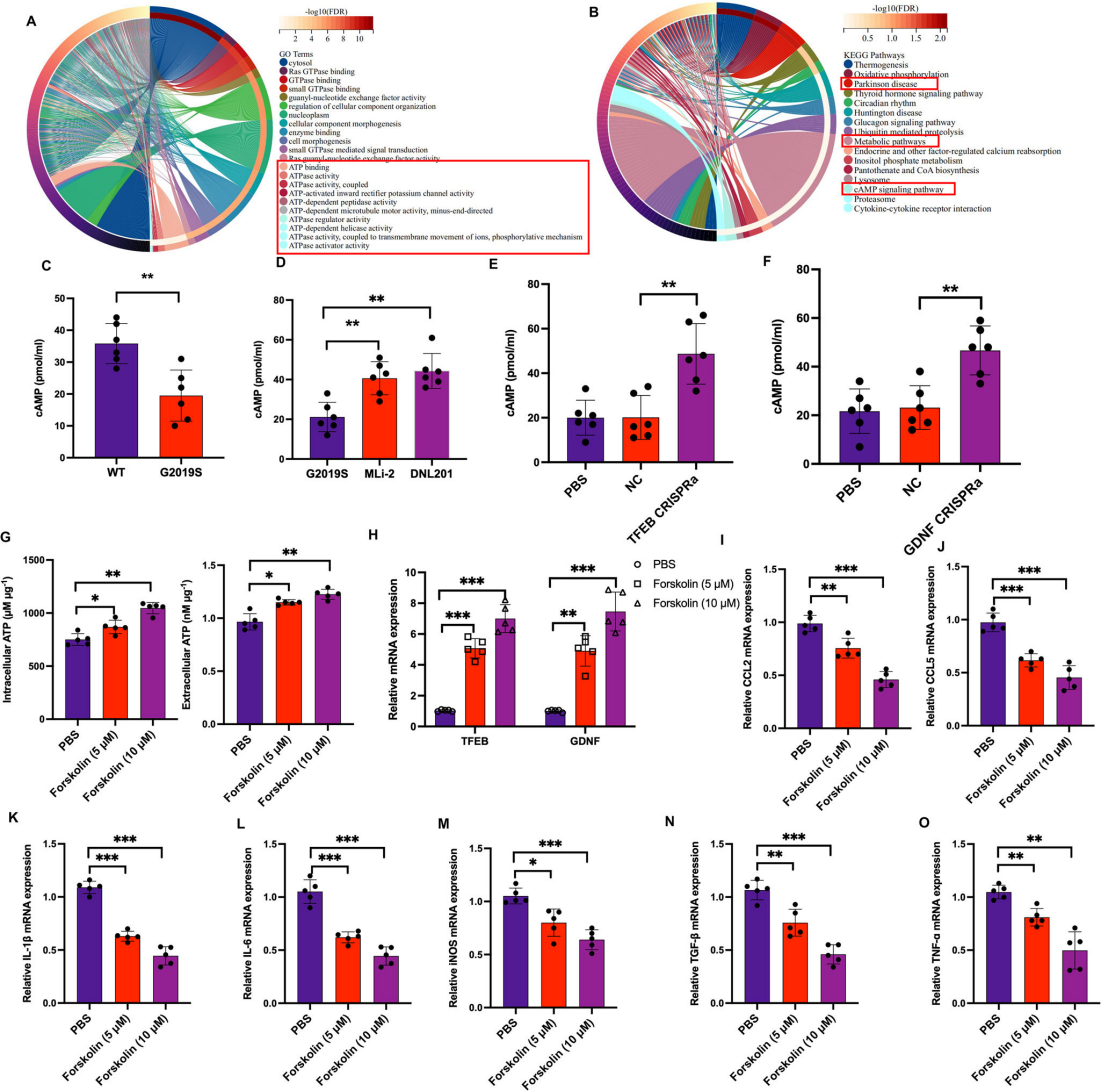
Disrupted cAMP–ATP coupling in LRRK2 G2019S and its rescue via the TFEB/GDNF (A,B) Single‐cell DEG enrichments visualized as circos plots. (A) KEGG highlights pathways linked to cAMP signaling, calcium signaling, oxidative phosphorylation/ATP generation, neuroactive ligand–receptor interaction, and cytokine–cytokine receptor interaction. (B) GO terms converge on processes related to ATP metabolism, adenylyl cyclase activity/cAMP biosynthesis, ion transmembrane transport, calcium homeostasis, membrane potential, and synaptic signaling (selected terms boxed). (C–F) cAMP measurements via ELISA. (C) WT versus G2019S; (D) effect of LRRK2 kinase inhibition (MLi‐2, DNL201); (E) TFEB CRISPRa versus controls (PBS, NC); (F) GDNF CRISPRa versus controls. (G,H) Forskolin (adenylyl cyclase activator) dose response. (G) Intracellular and extracellular ATP after PBS, 5 µm, or 10 µm forskolin. (H) TFEB and GDNF mRNA expression were measured by RT‐qPCR under the same conditions. (I,K–O) Inflammatory transcripts after forskolin (PBS, 5 µm, 10 µm): CCL2 and CCL5 (I); IL‐1β (K), IL‐6 (L), iNOS (M), TGF‐β1 (N), TNF‐α (O) were measured by RT‐qPCR. Data are shown as violin plots; *n* = 6 biologically independent samples per group. Statistics: two‐tailed *t*‐test for (C); one‐way ANOVA with Tukey post hoc for multi‐group panels (D–F, G–O). Significance: ** *p* < 0.01; *** *p* < 0.001; ns = not significant.

Single‐cell transcriptomic analysis identified 507 differentially expressed genes (Table S1). Gene Ontology (GO) enrichment converged on processes related to ATP metabolism, adenylyl cyclase activity, regulation of cAMP biosynthesis/signaling, ion transmembrane transport, calcium homeostasis, membrane potential, and synaptic signaling (Figure 7A). KEGG pathways likewise highlighted cAMP signaling, calcium signaling, neuroactive ligand–receptor interaction, oxidative phosphorylation/ATP generation, and cytokine–cytokine receptor interaction (Figure 7B), indicating perturbed cAMP–ATP coupling and neuroimmune signaling.

Guided by these enrichments, we quantified second messengers. cAMP was significantly reduced in G2019S cells versus WT (Figure 7C). Pharmacologic LRRK2 inhibition (MLi‐2 or DNL201) restored cAMP toward WT levels (Figure 7D). CRISPRa activation of TFEB or GDNF increased cAMP relative to non‐targeting control (Figure 7E,F), linking the TFEB/GDNF axis to cAMP production. To test whether adenylyl cyclase stimulation rescues bioenergetics and inflammation, we treated primary hippocampal astrocytes with forskolin, which can highly promote cAMP levels (Figure S10), which dose‐dependently elevated intracellular and extracellular ATP (Figure 7G) and upregulated TFEB and GDNF mRNA (Figure 7H). In parallel, forskolin reduced chemokine/prostaglandin inflammatory transcripts (CCL2, CCL5) (Figure 7I) and broadly suppressed pro‐inflammatory mediators (IL‐1β, IL‐6, iNOS2, TGF‐β1, and TNF‐α) (Figure 7K–O). To test GDNF dependence, we first silenced GDNF in astrocytes using CRISPR interference (CRISPRi) for 48 h and then modulated TFEB using CRISPRa or CRISPRi for a further 48 h. Notably, intracellular and extracellular cAMP and ATP levels remained stable across conditions (Figure S14B–D). Collectively, these data show that LRRK2–TFEB/GDNF signaling controls cAMP and cellular ATP, and that pharmacologic cAMP elevation mitigates the associated inflammatory program.

### 3.8 Altered TFEB Expression/Phosphorylation in the Hippocampus of LRRK2 G2019S Mice

**FIGURE 8:**
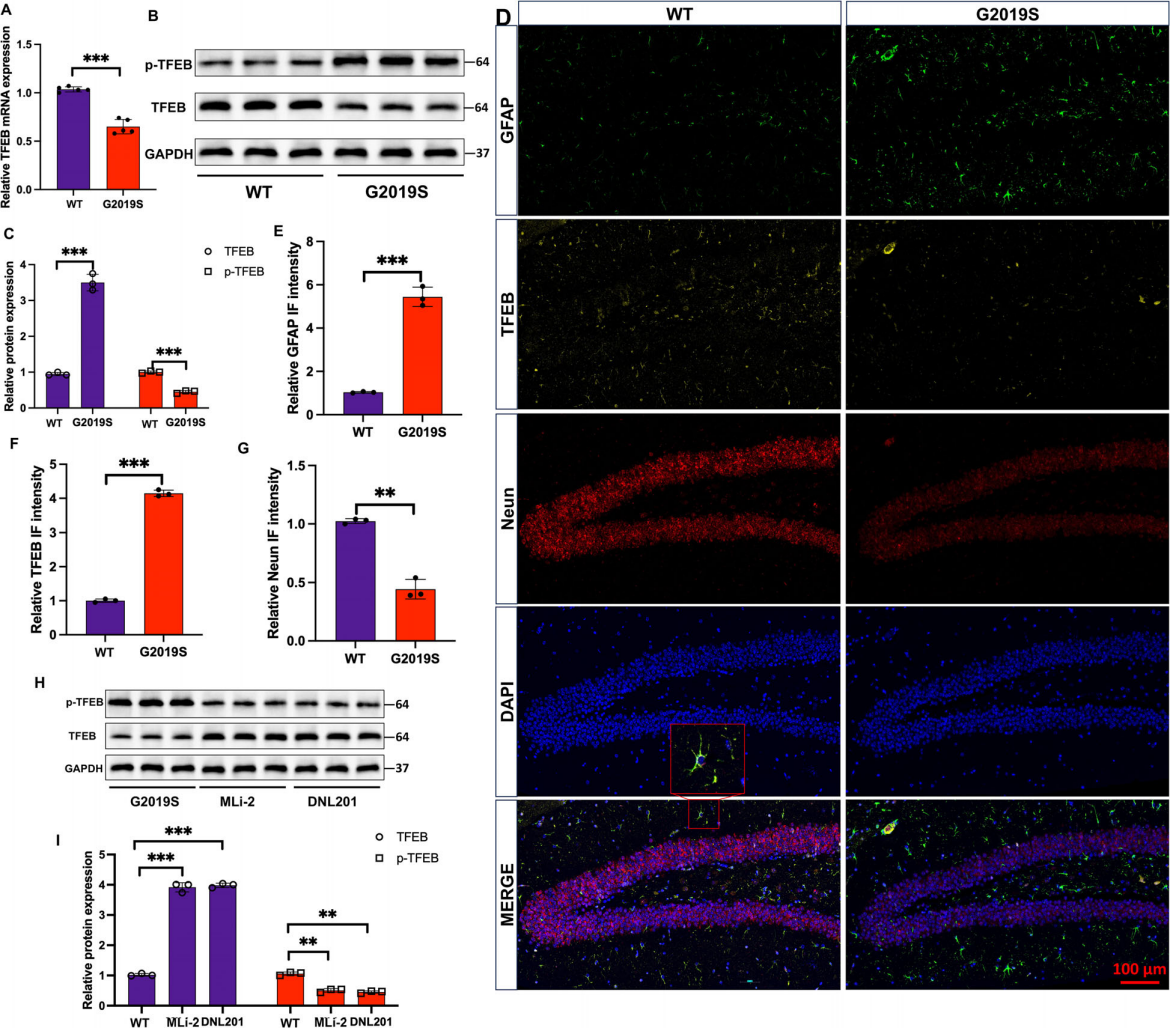
Altered TFEB expression/phosphorylation in the hippocampus of LRRK2 G2019S mice. (A) RT‐qPCR quantification of TFEB mRNA in hippocampi from WT and LRRK2 G2019S mice. (B,C) Western blot assessment of TFEB and p‐TFEB (B) with relative protein levels calculated (C). (D–G) Confocal immunofluorescence in hippocampus: TFEB, NeuN, and GFAP signals in WT and LRRK2 G2019S mice (D). Color code: green = anti‐GFAP, red = anti‐NeuN, yellow = anti‐TFEB. Scale bar = 50 µm. Quantification of GFAP (E), TFEB (F), and NeuN (G) fluorescence intensity. (H,I) TFEB and p‐TFEB protein levels re‐evaluated by western blot following MLi‐2 and DNL201 treatment. Replicates: western blot *n* = 3; others *n* = 5. Statistical annotations: * *p* < 0.05; ** *p* < 0.01; *** *p* < 0.001; ns = not significant.

Using PCR and western blot, we profiled TFEB in the hippocampus of LRRK2 G2019S mice and observed a substantial decrease in TFEB expression with a concomitant increase in phosphorylation, consistent with our in vitro observations (Figure 8A–C). To define cellular localization, fluorescent triple labeling was applied. TFEB signals were preferentially detected in astrocytes rather than neurons, and the G2019S mutation markedly diminished astrocytic TFEB in the hippocampus (Figure 8D–G). This reduction occurs despite augmented astrocyte activation and aligns with higher neuronal death, emphasizing a central role for astrocytic TFEB in hippocampal vulnerability. To test pharmacologic modulation in vivo, LRRK2 kinase activity was targeted in G2019S mice. Administration of the LRRK2 kinase inhibitors MLi‐2 and DNL201 produced a robust increase in TFEB expression together with a pronounced reduction in TFEB phosphorylation (Figure 8H,I; Figure S7). The decrease in phosphorylation was evident following inhibitor treatment (Figure 8H,I).

### 3.9 Hippocampal TFEB/GDNF Knockdown Exacerbates Depression‐Like Behaviors in Wild Type Mice

**FIGURE 9:**
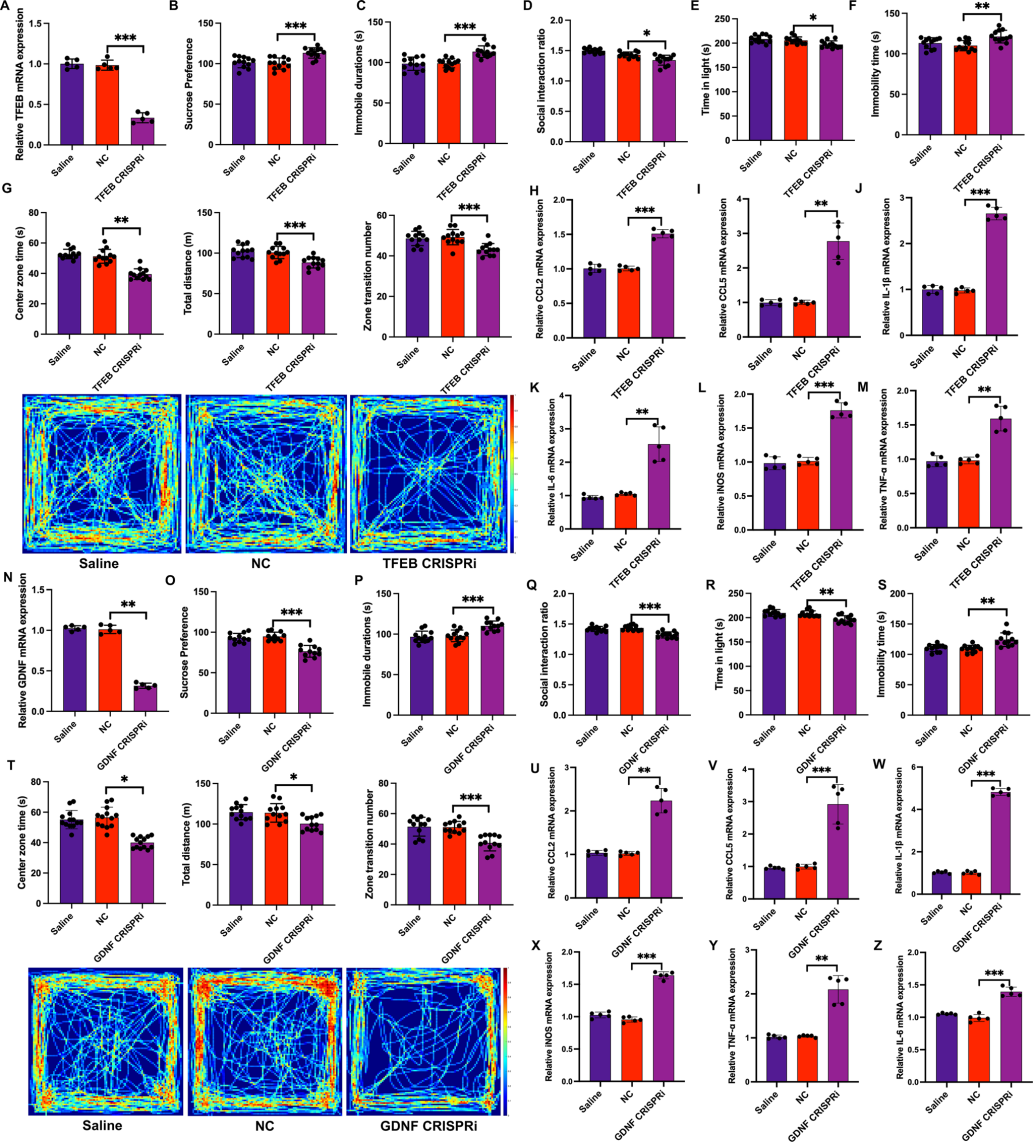
Hippocampal TFEB/GDNF knockdown exacerbates depression‐like behaviors in wild‐type mice. Hippocampal CRISPRi knockdown was used to probe the roles of TFEB and GDNF in WT mice. TFEB knockdown: (A) RT‐qPCR verification of TFEB mRNA reduction. (B–G) Depression‐related assays: SPT (B), FST (C), SIT (D), LDT (E), TST (F), and OFT (G); (G) also shows representative heat maps of time allocation in the open‐field arena. (H–M) RT‐qPCR for inflammatory transcripts normalized to GAPDH: CCL2 (H), CCL5 (I), IL‐1β (J), IL‐6 (K), iNOS (L), TNF‐α (M). GDNF knockdown: (N) RT‐qPCR confirmation of reduced GDNF mRNA. (O–T) Behavioral battery: SPT (O), FST (P), SIT (Q), LDT (R), TST (S), OFT (T) with representative heat maps in (T). (U–Z) RT‐qPCR of CCL2 (U), CCL5 (V), IL‐1β (W), IL‐6 (X), iNOS (Y), TNF‐α (Z), normalized to GAPDH. Replicates: behavioral tests *n* = 12; RT‐qPCR *n* = 5. Statistics: one‐way ANOVA with Tukey's post hoc test. Data: mean ± SD; significance denoted as * *p* < 0.05, ** *p* < 0.01, *** *p* < 0.001.

To test the roles of TFEB and GDNF in depressive phenotypes, CRISPR plasmids were used to downregulate each gene specifically in the hippocampus of WT mice (Figure 9A,N; Figure S8). Behavioral readouts showed a clear depressive‐like profile after knockdown: immobility increased in FST and TST (Figure 9C,F,P,S); sucrose preference declined in SPT (Figure 9B,O); social interaction decreased in SIT (Figure 9D,Q); and OFT revealed reduced center time, total distance, and zone transitions (Figure 9G,T). LDT further showed less time in the light compartment (Figure 9E,R). Together, hippocampal suppression of TFEB or GDNF induced despair‐like behavior, anhedonia, social withdrawal, and anxiety across assays. Building on the link between TFEB, GDNF, and astrocytic state, we assessed hippocampal inflammation following gene knockdown. Suppression of either TFEB or GDNF produced a significant decrease in CCL2, CCL5, IL‐1β, TGF‐β1, IL‐6, iNOS, and TNF‐α (Figure 9H–M,U,V).

### 3.10 Hippocampal TFEB Drives GDNF Upregulation and Behavioral Rescue in LRRK2 G2019S Mice

**FIGURE 10:**
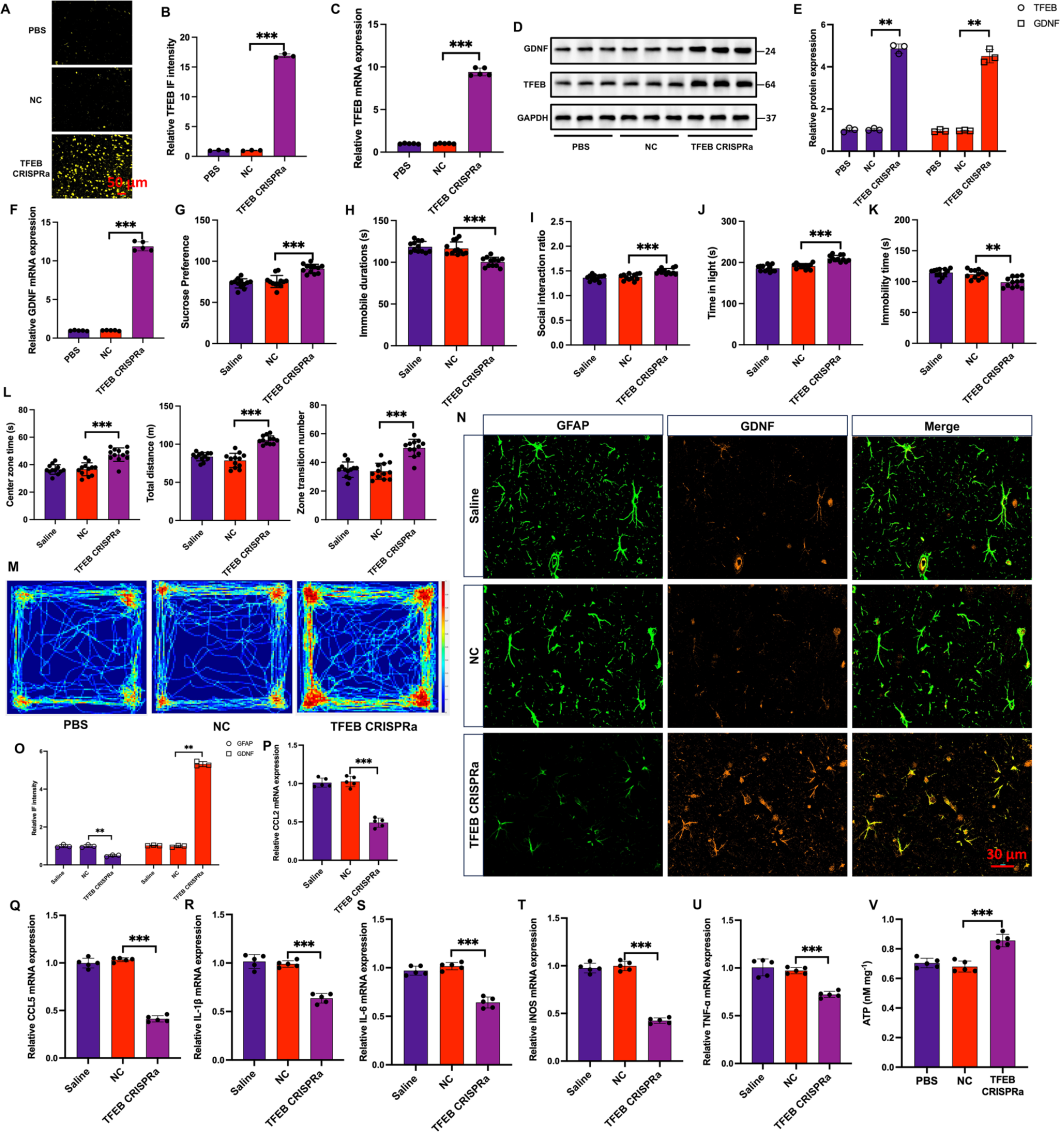
Hippocampal TFEB drives GDNF upregulation and behavioral rescue in LRRK2 G2019S mice. (A–F) Following hippocampal TFEB CRISPRa delivery, confocal immunofluorescence visualized TFEB (A; yellow = anti‐TFEB; scale bar = 50 µm) with relative fluorescence intensity quantified (B). RT‐qPCR verified TFEB mRNA in the hippocampus (C). Western blot assessed TFEB and GDNF proteins (D) with relative expression calculated (E). RT‐qPCR also confirmed GDNF mRNA (F). (G–L, M) Depression‐related behavioral battery post‐TFEB CRISPRa: SPT (G), FST (H), SIT (I), LDT (J), TST (K), OFT (L). (M) shows representative heat maps of time allocation within the open‐field arena. (N,O) Confocal immunofluorescence for GDNF and GFAP in the hippocampus after injections (N; green = anti‐GFAP, red = anti‐GDNF; scale bar = 30 µm), with relative fluorescence intensities quantified (O). (P–U) RT‐qPCR of inflammatory transcripts normalized to GAPDH: CCL2 (P), CCL5 (Q), IL‐1β (R), IL‐6 (S), iNOS (T), TNF‐α (U). (V) ATP levels measured in media from hippocampal slices. Data presentation: mean ± SD. Replicates: behavioral tests *n* = 12; western blot *n* = 3; others *n* = 5. Statistics: one‐way ANOVA with Tukey's multiple comparisons. Significance: * *p* < 0.05; ** *p* < 0.01; *** *p* < 0.001.

Building on our in vitro findings, we activated TFEB in the hippocampus of LRRK2 G2019S mice using CRISPR plasmids. Successful activation was verified by immunofluorescence staining, PCR, and western blot (Figure 10A–E). In vivo, TFEB activation increased GDNF (Figure 10D–F), reinforcing TFEB as a regulator of GDNF transcription with downstream effects on astrocytic function and neuroprotection. Behaviorally, TFEB activation produced broad improvements: immobility decreased in FST and TST (Figure 10H,J), sucrose preference rose in SPT (Figure 10G), and social interaction time increased in SIT (Figure 10I). In OFT, mice showed more center time, greater total distance, and more zone transitions (Figure 10L,M); in LDT, time in the light compartment was extended (Figure 10J). Extending these analyses, hippocampal TFEB activation attenuated astrocyte activation and further elevated GDNF within astrocytes (Figure 10N,O). It also rescued neuronal death induced by G2019S (Figure S9), highlighting a dual action of TFEB—enhancing neurotrophic support while restraining astrocytic activation. In parallel, TFEB activation suppressed inflammatory factors in the hippocampus that were elevated by the mutation (Figure 10P–U) and restored ATP in astrocytes (Figure 10V), and cAMP (Figure S13), indicating a coordinated impact on inflammatory tone and cellular energy.

To assign the rescue effects to astrocytes in vivo, we expressed GDNF under an astrocyte‐enriched promoter (AAV‐gfaABC1D‐GDNF) via a single bilateral stereotaxic injection into the hippocampus. Immunofluorescence confirmed that GDNF signal in the injected region was predominantly detected in GFAP‐positive cells (Figure S15A). Functionally, AAV‐gfaABC1D‐GDNF produced consistent improvements across multiple behavioral readouts. Compared with NC, mice receiving AAV‐gfaABC1D‐GDNF showed increased sucrose preference in SPT (Figure S15B), reduced immobility duration in FST (Figure S15C), increased social interaction ratio in SIT (Figure S15D), and increased time spent in the light compartment in LDT (Figure S15E).

### 3.11 ATP/cAMP Administration Improves G2019S‐Induced Depression

**FIGURE 11:**
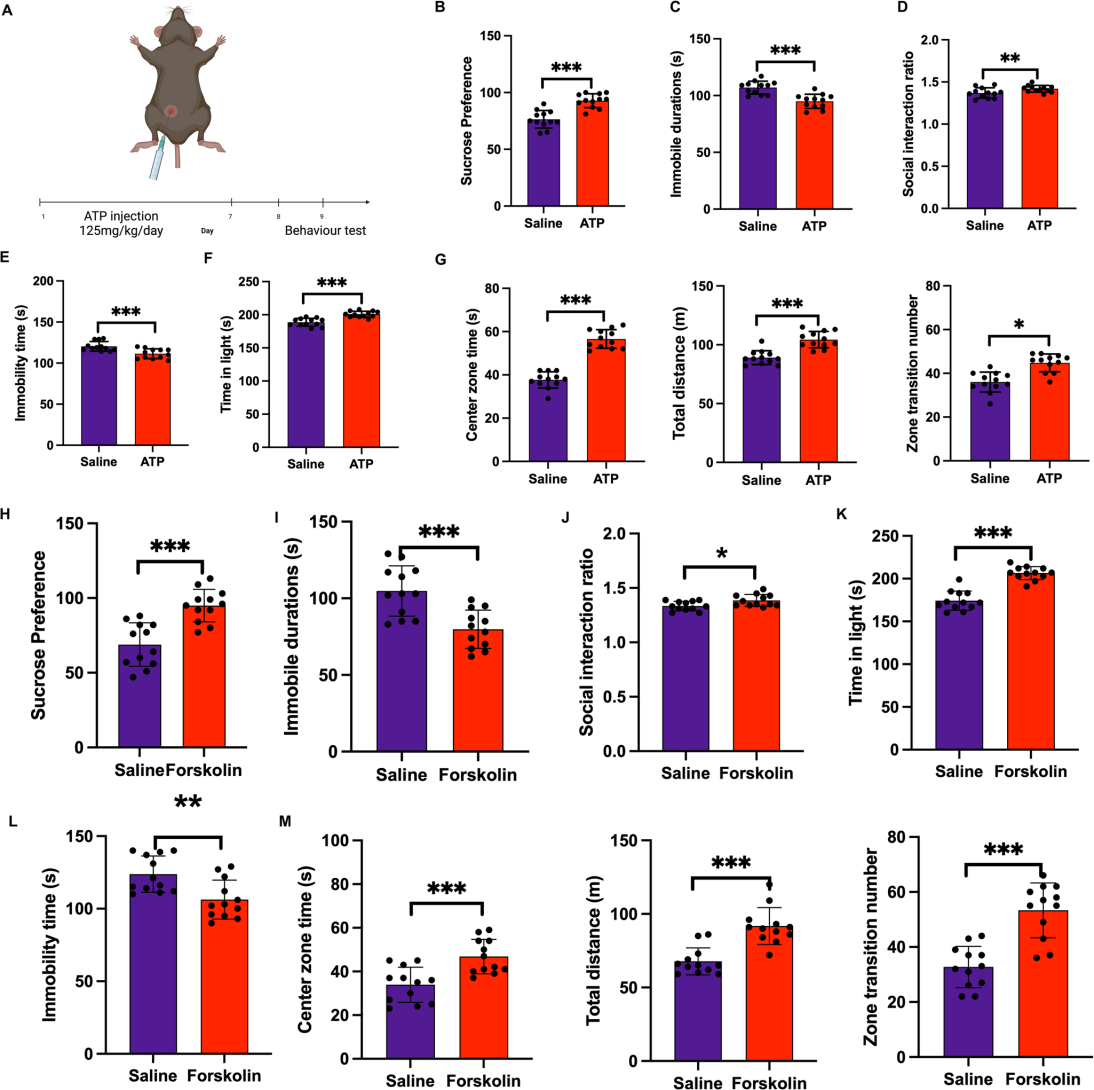
ATP/cAMP administration improves G2019S‐induced depression. (A) Schematic of bilateral hippocampal microinjection in 20‐week‐old LRRK2 G2019S mice (ATP 25 µm, 1 µL, 0.2 µL/min). ATP supplementation (saline vs. ATP): (B–F) Depression‐related assays—SPT sucrose preference (B), FST immobile duration (C), SIT social‐interaction ratio (D), LDT time in light (E), and TST immobility (F). (G–I) OFT indices—center‐zone time, total distance, and zone‐transition number. cAMP elevation (saline vs. forskolin): (H–L) Behavioral outcomes with adenylyl cyclase activation (forskolin): SPT (H), FST (I), SIT (J), LDT (K), TST (L). (M–O) OFT metrics—center‐zone time, total distance, and zone transitions. Data are shown as violin plots; values are mean ± SD. Sample size: *n* = 12 biologically independent mice per group for all behavioral panels. Statistics were performed with two‐tailed tests as appropriate. Significance: * *p* < 0.05; ** *p* < 0.01; *** *p* < 0.001; ns = not significant.

Prompted by the ATP deficit observed in the brains of LRRK2 G2019S mice, we tested whether direct replenishment improves behavior (paradigm in Figure 11A; Figure S11A). Mice received ATP 25 mg/kg/day by bilateral hippocampus injection, assessed 4 h later, and ATP 125 mg/kg/day i.p. for 7 days, and then behavioral testing was conducted on day 9. Across assays, ATP produced robust benefits: immobility fell in FST and TST (Figure 11C–E; Figure S11C–E); sucrose preference increased in SPT (Figure 11B; Figure S11B); and social interaction time rose in SIT (Figure 11D; Figure S11D). In the OFT, treated mice spent more time in the central zone, traveled greater distances, and made more zone transitions (Figure 11G; Figure S11G). The LDT likewise showed longer time in the light compartment (Figure 11F; Figure S11F). Together, these results indicate that ATP supplementation mitigates the depression‐like phenotype associated with G2019S.

To verify the direct antidepressant effects of cAMP in the hippocampus, we conducted 0.2 µL forskolin targeted microinjections into the bilateral hippocampus of LRRK2 G2019S mutant mice, followed by behavioral assessments 6 h post‐infusion. Hippocampal microinjection of forskolin significantly attenuated LRRK2 G2019S–associated depressive‐like behaviors by elevating hippocampal cAMP levels (Figure S12). Notable outcomes included a decreased immobility time in both FST and TST (Figure 11J,M), increased sucrose preference in SPT (Figure 11I), enhanced social interaction time, and extended duration spent in the light compartment during the LDT (Figure 11K,L). In the OFT, the mice treated with ATP spent more time in the central zone, covered greater distances, and made more transitions between zones (Figure 11N). These behavioral improvements confirm that direct ATP/cAMP supplementation to the hippocampus can effectively counteract depressive behaviors induced by the LRRK2 G2019S mutation.

## 4 Discussion

The LRRK2 G2019S variant sits at the intersection of genetics and clinical expression in PD [2, 6]. Beyond its established contribution to motor manifestations, this mutation aligns with a wider array of non‐motor features—most notably depression, a major determinant of quality of life [2, 6, 33]. Framing neuropsychiatric symptoms through the lens of this genotype helps bridge molecular mechanisms with comprehensive PD management [43]. Here, we define a pathway whereby LRRK2 G2019S constrains TFEB, resulting in aberrant GDNF transcription; this, in turn, drives astrocytic inflammatory activation and disrupts ATP homeostasis—two convergent processes that precipitate depression‐like behaviors in PD.

We observed a clear age‐dependent emergence of depression‐like behaviors in LRRK2 G2019S mice, with differences becoming evident at ∼20 weeks, aligning with the progressive trajectories typical of neurodegenerative disorders. The lag before behavioral divergence implies that G2019S initiates subtle, cumulative perturbations that cross a threshold later in life. Although individual behavioral assays may be influenced by changes in general activity, the depressive‐like phenotypes described here were assessed using a battery of complementary tests, supporting alterations in affective state rather than overt motor impairment. This is salient for PD, where non‐motor features—including depression—can precede classic motor signs, suggesting a window for early intervention against incipient mechanisms [44]. Consistent with models of PD depression, converging pathology in mood‐regulatory circuitry—particularly PFC and hippocampus—is implicated [45]. Prior work highlights astrocytes as key custodians of neuronal viability and neuroinflammatory tone [12], and astrocytic dysfunction has been increasingly tied to depressive pathophysiology [20]. At present, dedicated studies on the role of astrocytes in the onset and progression of PD‐related depression remain scarce, and the specific mechanisms are even less well defined [46]. In our data, both PFC and hippocampus were affected, but the hippocampus showed greater vulnerability, with stronger neuroinflammatory injury and neurotrophic factor deficits, paralleling more pronounced neurodegeneration than in PFC. The accompanying astrocyte activation and elevated inflammatory mediators in the G2019S hippocampus point to astrocytes as central drivers of the depressive phenotype, consistent with a mutation‐triggered inflammatory program that promotes neuronal death and thereby fuels depression‐like behaviors.

In the LRRK2 G2019S–induced depression model, we identify a central role for the LRRK2 G2019S mutation in controlling the transcription factor TFEB within astrocytes. TFEB functions as a master regulator of autophagy and lysosomal biogenesis—processes essential for cellular homeostasis [16, 42]. Specifically, the results show that G2019S significantly reduces TFEB expression in astrocytes and, beyond expression, that LRRK2 physically associates with TFEB and phosphorylates it. Using molecular docking in combination with phosphorylation assays, we mapped four common phosphorylation sites on TFEB and demonstrated that LRRK2 selectively targets Ser211. Notably, Ser211 is a well‐established regulatory phosphorylation site on TFEB. Phosphorylation at Ser211 correlated with suppressed TFEB transcriptional activity, consistent with prior work showing that TFEB phosphorylation impairs nuclear translocation [47, 48]. Also, the studies showed that mTORC1‐dependent phosphorylation at Ser211 promotes 14‐3‐3 binding and cytosolic retention of TFEB, thereby suppressing TFEB transcriptional activity under nutrient‐replete conditions [49]. Our identification of LRRK2 G2019S–associated TFEB Ser211 phosphorylation therefore suggests that a PD‐linked kinase can converge on this canonical TFEB “localization switch”, potentially providing a disease‐relevant input that interfaces with the mTORC1–TFEB regulatory framework [49]. In line with this concept, recent work has implicated LRRK2 kinase activity in suppressing MiT/TFE family nuclear localization and lysosome gene programs in immune‐lineage CNS cells, supporting a broader link between LRRK2 signaling and MiT/TFE‐controlled homeostasis [39]. Concordantly, nuclear–cytoplasmic separation experiments revealed a marked reduction in nuclear TFEB in LRRK2 G2019S astrocytes. This impaired nuclear entry diminishes TFEB's capacity to drive gene programs supporting neurotrophic signaling and cellular equilibrium—most notably the regulation of GDNF—thereby linking LRRK2–TFEB signaling to astrocytic dysfunction in this context.

A consistent downstream consequence of LRRK2 G2019S across our datasets is a pronounced shortfall of GDNF in the hippocampus [19]. As detailed above, this reduction arises chiefly from dampened TFEB‐driven transcription in astrocytes, rather than from neuronal changes, placing astrocytes at the center of the depression‐related pathology in this genotype. Given the well‐established neuroprotective role of GDNF in sustaining neuronal viability and function [19, 36], its loss creates conditions that favor neurodegeneration and behavioral impairment. Mechanistically, impaired TFEB activity curtails GDNF transcription and pushes astrocytes toward a neurotoxic secretory state, amplifying inflammatory signaling and coinciding with marked neuronal apoptosis in the hippocampus. Beyond inflammation, our data identify that the TFEB/GDNF axis is functionally linked to astrocytic energy handling. In G2019S mice, ATP homeostasis in astrocytes is broadly depressed—both intracellular production and extracellular release decline in parallel. Astrocyte‐derived ATP is indispensable for meeting neuronal energy needs [50], supporting synaptic plasticity, neurotransmission, and ion‐gradient maintenance that underlie neuronal firing [51, 52, 53]. Once released, ATP is enzymatically converted to adenosine in the extracellular space, providing an additional layer of neuromodulation [54]. This astrocyte–neuron energy coupling is especially critical in the hippocampus, a hub for mood and cognitive processes implicated in depression [20]. Consistent with this view, raising cAMP with the adenylyl cyclase activator forskolin elevated astrocytic ATP and tempered inflammatory readouts, while exogenous ATP supplementation stabilized the local energy landscape; both manipulations improved depression‐like behaviors in vivo—supporting a model in which second‐messenger tone (cAMP) and energetic sufficiency (ATP) are jointly impaired and jointly rescuable. Astrocytic cAMP–PKA signaling has been increasingly recognized as a key coordinator of brain energy metabolism, linking neuromodulatory cues to astrocyte metabolic activation and brain‐wide energetic homeostasis [55]. In line with this framework, our data support positioning cAMP/ATP and cytokine outputs downstream of a TFEB→GDNF node, based on the GDNF‐dependence/occlusion experiments and pathway‐targeted rescues. TFEB overexpression (in vivo/in vitro) increased GDNF, dampened astrocyte‐driven inflammation, corrected intra‐ and extracellular ATP deficits, and improved neuronal survival. In parallel, direct GDNF overexpression reproduced these benefits, stabilizing inflammatory markers and normalizing ATP levels within hippocampal circuits. Together with the GDNF‐dependence/occlusion experiments, these downstream bypass rescues support a TFEB→GDNF→(cAMP/ATP and inflammatory outputs) ordering rather than a purely correlative association. Taken together, the findings argue that LRRK2 G2019S → TFEB/GDNF dysregulation deranges a cAMP–ATP axis in astrocytes; restoring either the transcriptional node (TFEB/GDNF) or the downstream messengers (cAMP/ATP) rebalances astrocyte–neuron support and mitigates depression‐like phenotypes.

Targeting LRRK2 kinase activity emerges from our data as a tractable strategy for countering the LRRK2 G2019S phenotype. Inhibitor studies with MLi‐2 and DNL201 showed broad efficacy: beyond dampening depression‐like behaviors, they corrected upstream regulatory defects we identified. Specifically, kinase blockade reinstated TFEB expression and lowered TFEB phosphorylation, thereby facilitating TFEB nuclear entry and enhancing GDNF transcription. The resultant rise in GDNF aligned with improved neuronal health and paralleled the behavioral rescue observed in PD models. At the cellular level, both compounds curbed astrocyte activation and attenuated hippocampal neuroinflammation, addressing core features of the astrocytic pathology. Crucially, these effects extended to bioenergetic and second‐messenger signaling. Inhibitor treatment improved ATP synthesis/availability in the hippocampus, providing a mechanistic basis for greater neuronal resilience. In parallel, we observed evidence for repaired cAMP–ATP coupling: by relieving LRRK2‐dependent suppression of the TFEB/GDNF axis, kinase inhibition elevated cAMP tone (consistent with enhanced adenylyl cyclase signaling) and, downstream, normalized astrocytic ATP production and release. Together, these changes—TFEB de‐phosphorylation and nuclear translocation, GDNF upregulation, reduced astrocytic reactivity, restored cAMP/ATP homeostasis—offer a coherent explanation for the behavioral improvement under MLi‐2 and DNL201, and highlight LRRK2 kinase inhibition as a convergent therapeutic lever for LRRK2 G2019S–associated depression.

Our findings position TFEB and GDNF as upstream gatekeepers of astrocytic homeostasis whose disruption propagates to cAMP/ATP signaling and, ultimately, to behavior. In wild‐type mice, hippocampal knockdown of TFEB or GDNF was sufficient to yield a depressive‐like profile—greater immobility in FST/TST, reduced sucrose preference in SPT, diminished exploratory performance in OFT, and lower social interaction—indicating that both factors are necessary to sustain normal affective tone. In the LRRK2 G2019S context, therapeutically increasing downstream messengers reversed these deficits: ATP supplementation improved behavior whether delivered intraperitoneally or via bilateral hippocampal injection, and elevating cAMP likewise ameliorated depressive‐like readouts. Together, these observations support a model in which a TFEB → GDNF → cAMP/ATP cascade stabilizes astrocyte–neuron support; perturbation (TFEB/GDNF loss) drives depressive‐like behaviors, whereas restoring cAMP and/or ATP rebalances bioenergetics and circuit function, highlighting tractable metabolic entry points for intervention.

Several limitations should be noted. First, although our data support an astrocyte‐centered TFEB–GDNF–cAMP/ATP cascade, LRRK2 is expressed across multiple CNS cell types, and we cannot exclude additional contributions from neurons and microglia in vivo, including microglia‐driven inflammatory programs and intercellular crosstalk. Second, this study focuses on the kinase‐activating LRRK2 G2019S variant; whether other pathogenic LRRK2 mutations converge on the same pathway across cell types remains to be determined. Third, primary hippocampal astrocyte and neuron cultures used for mechanistic dissections were prepared from postnatal day 0–3 pups, and thus represent a reductionist system that does not fully recapitulate adult/aged brain physiology; accordingly, cumulative age‐dependent processes, potentially including cellular senescence, may contribute to the delayed emergence of depressive‐like behaviors observed in vivo. Fourth, all behavioral experiments were performed in male mice. Although the SIT used a perforated containment‐cage design to prevent direct dominance/subordinate encounters, male‐specific social hierarchy or stress‐related responses may still influence social investigation behavior and should be considered when interpreting reduced approach to an unfamiliar conspecific.

**FIGURE 12:**
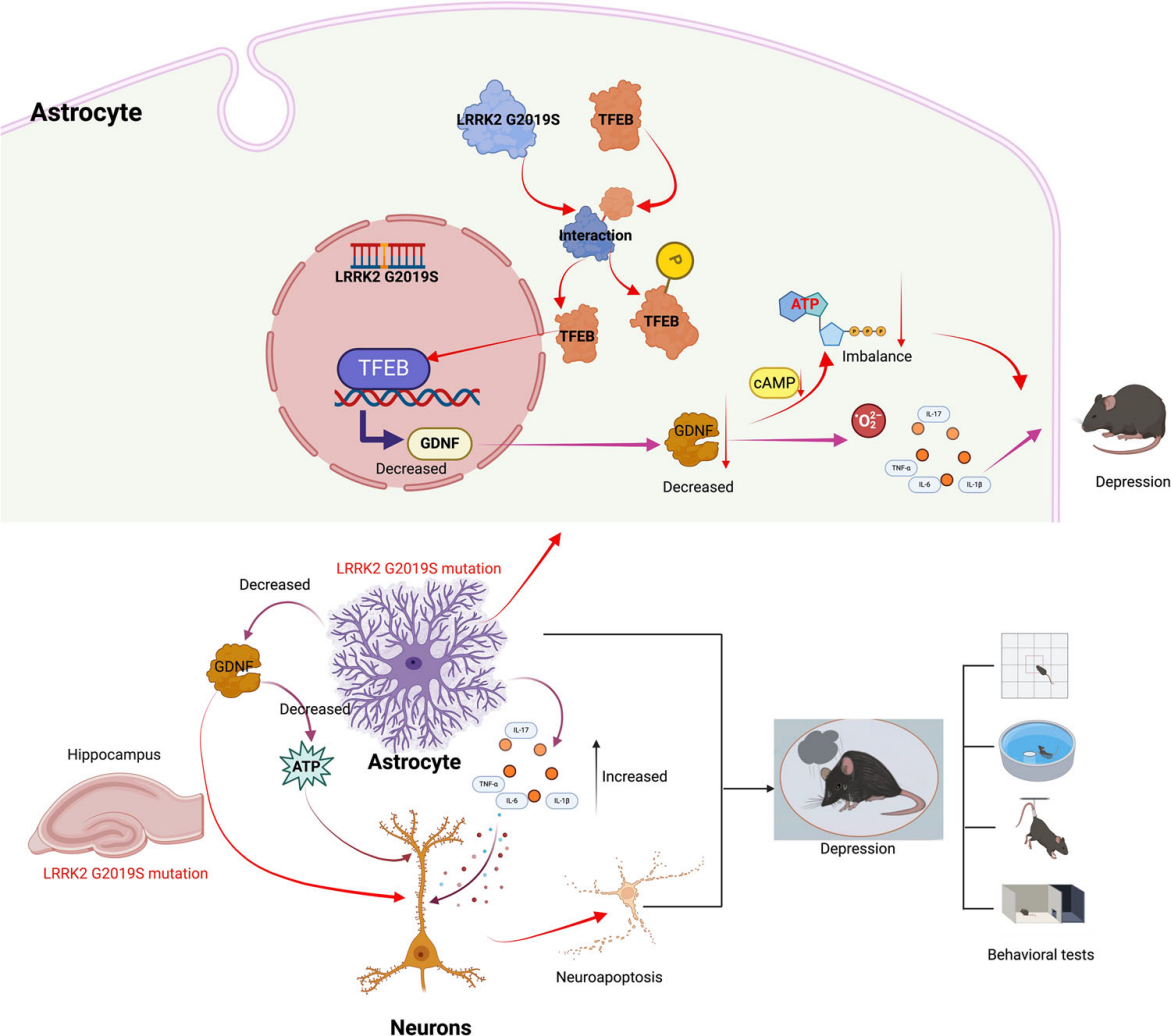
Schematic showing the mechanisms underlying LRRK2 G2019S‐induced astrocyte neurotoxicity in the hippocampus via interacting TFEB/GDNF leads to depressive alterations in PD.

In sum, LRRK2 G2019S emerges as a principal driver of age‐dependent depression‐like behaviors in PD, acting through neuronal apoptosis and astrocyte dysfunction—the latter characterized by neuroinflammation and an ATP imbalance. Mechanistically, the mutation inhibits TFEB expression, promotes TFEB phosphorylation, and directly binds TFEB, thereby disrupting GDNF transcription. This derailment initiates a cascade in which astrocytes adopt a pro‐inflammatory state and bioenergetic coupling deteriorates, reflected by deficits in both ATP and its upstream second messenger cAMP. Together, these changes provide a coherent substrate for the depressive phenotype. Therapeutically, our data highlight convergent points of intervention (Figure 12): LRRK2 kinase inhibition relieves TFEB repression/phosphorylation to restore GDNF, dampen astrocytic inflammation, and repair cAMP/ATP homeostasis; targeting TFEB/GDNF directly reconstitutes trophic support and normalizes astrocytic state; and supplementing ATP and elevating cAMP provides a metabolic bypass that stabilizes energy supply and improves behavior. These approaches address symptoms while acting on the underlying molecular circuitry, offering a more comprehensive path toward managing depression linked to neurodegenerative mutations.

## Author Contributions

Conceptualization and experimental design: Y.L.P., S.T., and Z.Y.F. Literature review, data analysis, and writing: Y.L.P. and S.T. PD mouse model, behavioral testing, and outcome analysis: Y.L.P. and H.M. Validation experiments: J.Q., K.S., and Z.Y.F. Supervision and critical revision of the manuscript: J.Q., Y.L.P., Z.Y.F., and S.T.

## Funding

This study was supported by the funds of the University Clinic Heidelberg.

## Conflicts of Interest

The authors declare no conflicts of interest.

## Supporting information




**Supporting File**: advs77059‐sup‐0001‐SuppMat.pdf.

## Data Availability

Research data are not shared.
