# Supplementary material for: Disrupted TFEB/GDNF–cAMP/ATP Coupling Underlies Astrocytic Dysfunction and Depression in LRRK2 G2019S Parkinson's Mice
Source: Adv Sci (Weinh). 2026 Sep 16:e22066. Online ahead of print. doi: 10.1002/advs.202522066 (PMC13583120; doi:10.1002/advs.202522066)
Supplement: Supplementary file 1 [file ADVS-9999-e22066-s001.pdf]

Supplementary Figures

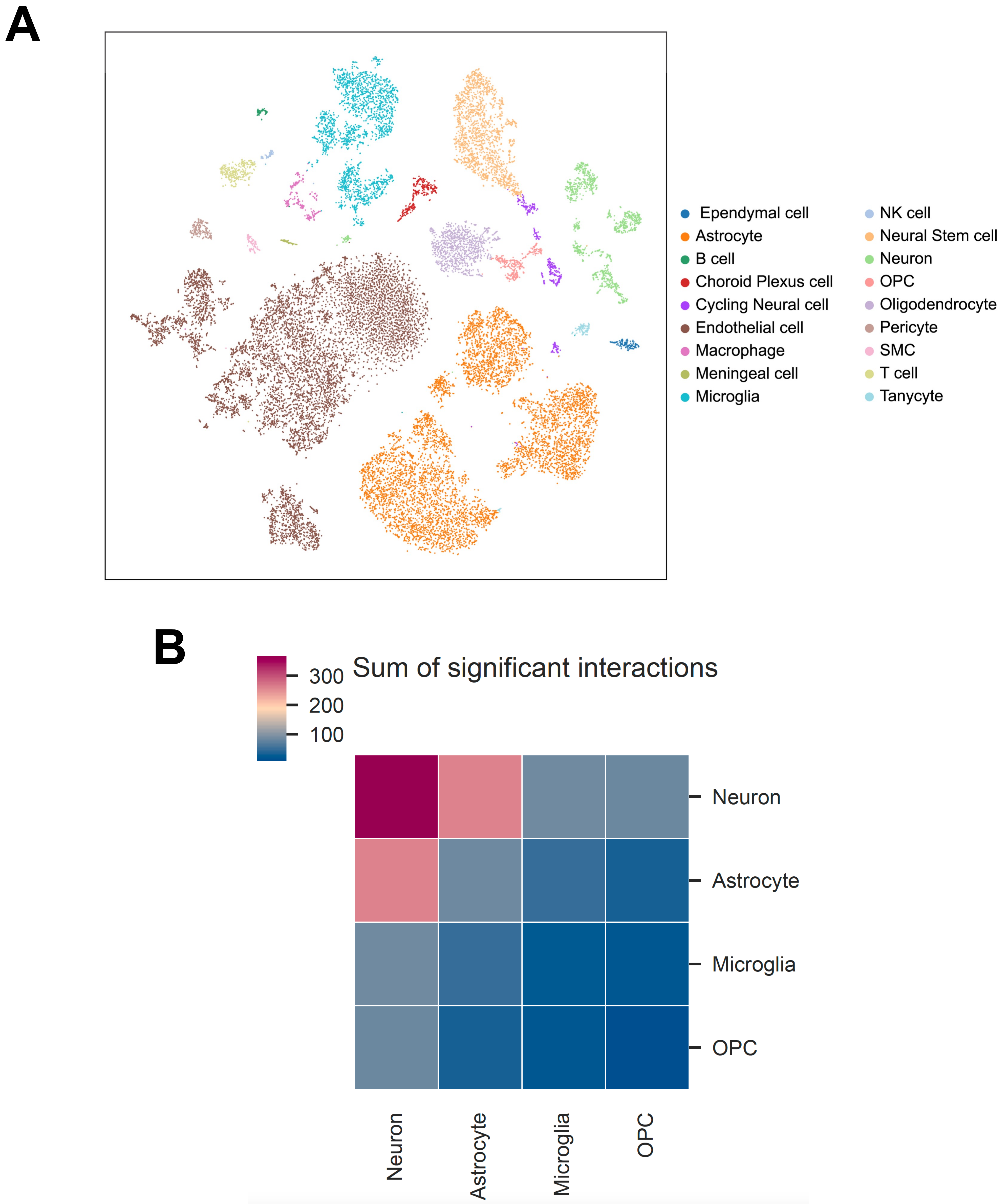

Supplementary Figure 1

Supplementary Figure 1. (A) The UMAP plot presents all sequenced cells based on cell type from the dataset. (B) The sum of significant interactions between four cell types: neurons, astrocytes, microglia, and OPCs (oligodendrocyte precursor cells). The color gradient from blue to red represents the increasing number of interactions, with blue indicating fewer interactions (around 100) and red indicating more interactions (up to 300). The matrix shows that neurons have the highest number of self-interactions (dark red), while interactions between other cell types, particularly those involving OPCs, are less frequent (blue). This analysis highlights the distinct interaction patterns between these cell types in the single-cell dataset.

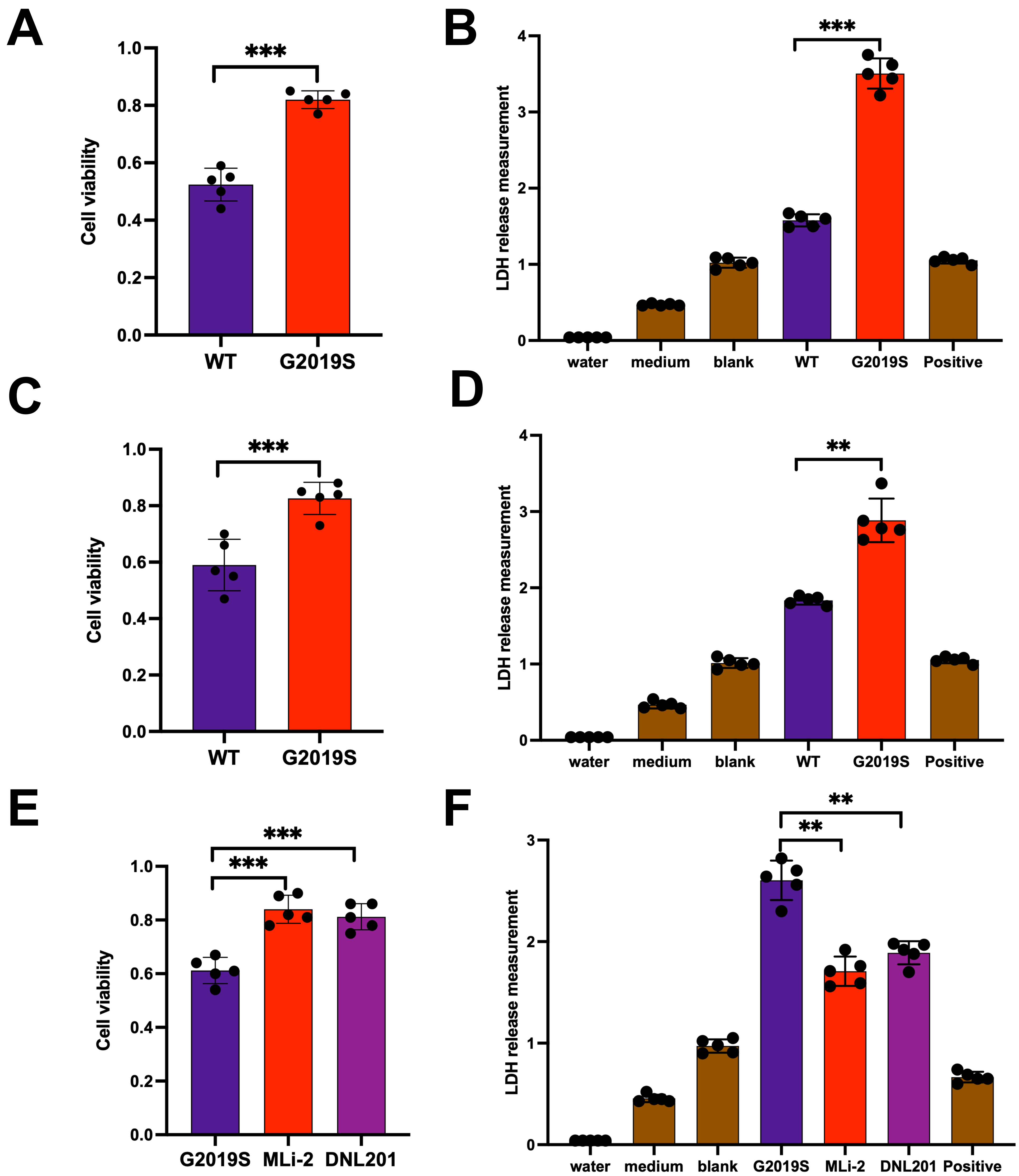

Supplementary Figure 2

Supplementary Figure 2. (A) Cell viability of primary neurons from wild and G2019S mutant mice was assessed using the CCK-8 assay. (B) Cell death of primary neurons from wild and G2019S mutant mice was determined by LDH assay. (C) After 48 hours of culture of primary astrocytes, we collected the conditioned medium and transferred it to wild primary neurons. After 48 hours of co-culture, cell viability of primary neurons was assessed using the CCK-8 assay. (D) After 48 hours of culture of primary astrocytes, we collected the conditioned medium and transferred it to wild primary neurons. After 48 hours of co-culture, cell viability of primary neurons was determined by LDH assay. (E-F) Primary G2019S astrocytes were treated by MLi-2 and DNL201 for 24h. Then we collected the conditioned medium and transferred it to wild primary neurons. After 48 hours of co-culture, cell viability of primary neurons was assessed using the CCK-8 assay. (F) Cell death was determined by LDH assay. Data were presented as means ± SD. The experiments were carried out 5 times (n=5). The difference in folds is statistically significant. $^{**}P < 0.01$, $^{***}P < 0.001$.

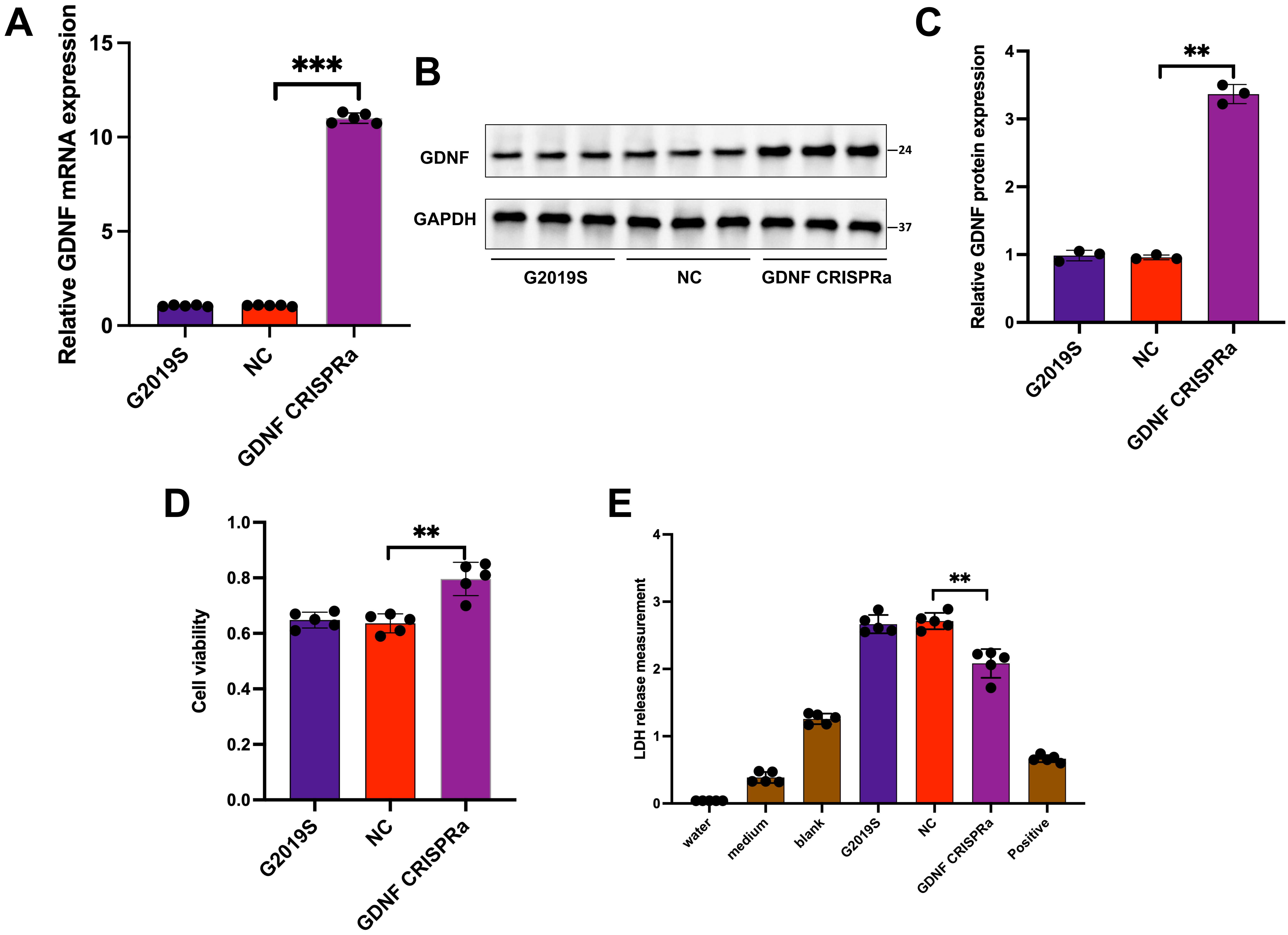

Supplementary Figure 3

Supplementary Figure 3. (A) The relative mRNA level of GDNF in the astrocytes was confirmed by RT-qPCR (n=5). (B-C) TFEB and GDNF protein expressions were assessed by western blot (B), with relative expression quantified (C) (n=3). (D) Cell viability was assessed using the CCK-8 assay (n=5). (E) Cell death was determined by LDH assay (n=5). Data were presented as means ± SD. The experiments were carried out 5 times. The difference in folds is statistically significant. $^{**}P < 0.01$, $^{***}P < 0.001$.

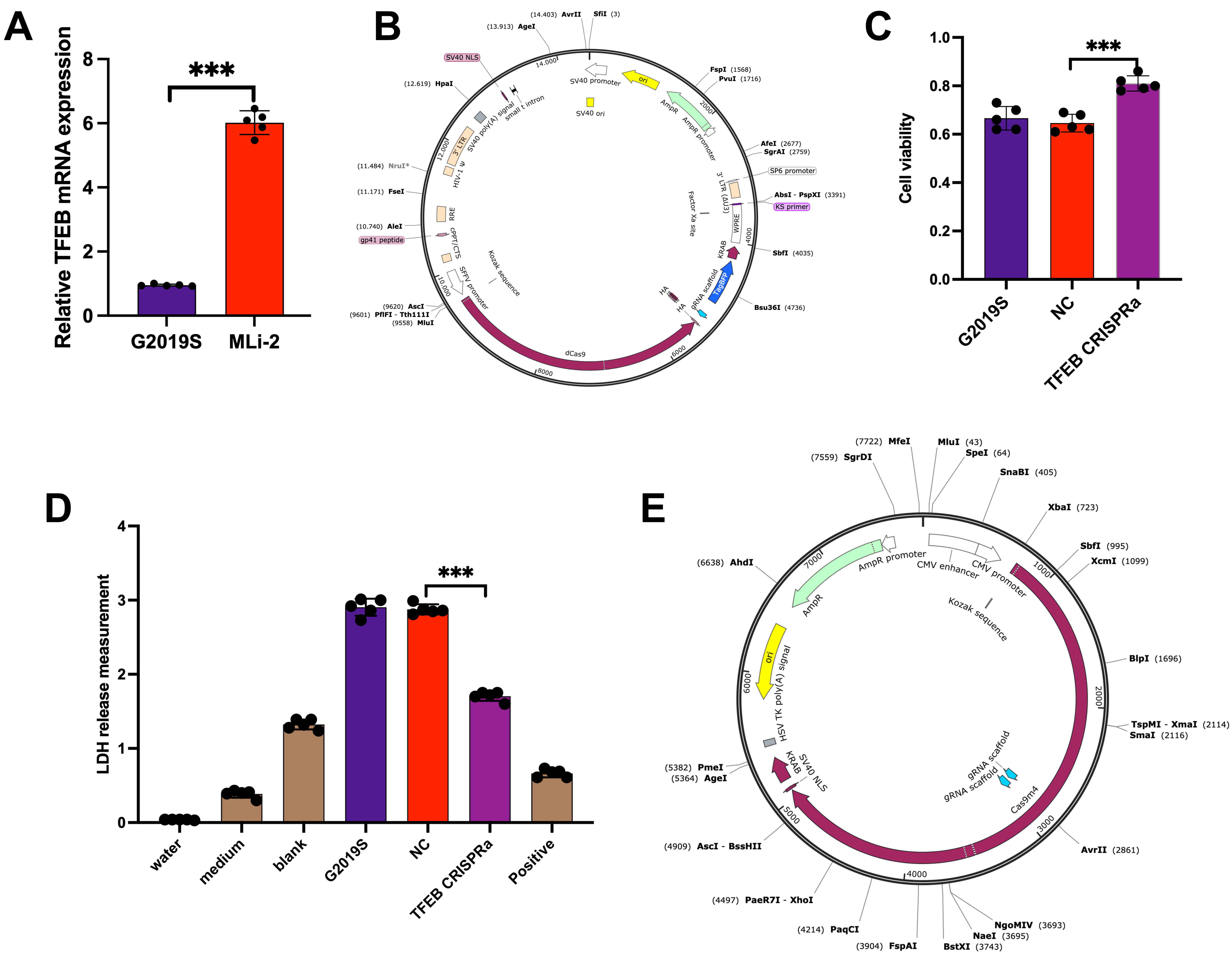

Supplementary Figure 4

Supplementary Figure 4. (A) The relative mRNA level of TFEB in the astrocytes was confirmed by RT-qPCR. (B) Schematic representation of TFEB CRISPRa plasmids. (B) Schematic representation of TFEB CRISPRa plasmids. (C) Cell viability was assessed using the CCK-8 assay. (D) Cell death was determined by LDH assay. (E) Schematic representation of GDNF CRISPRi plasmids. Data were presented as means ± SD. The experiments were carried out 5 times (n=5). The difference in folds is statistically significant. ****P* < 0.001.

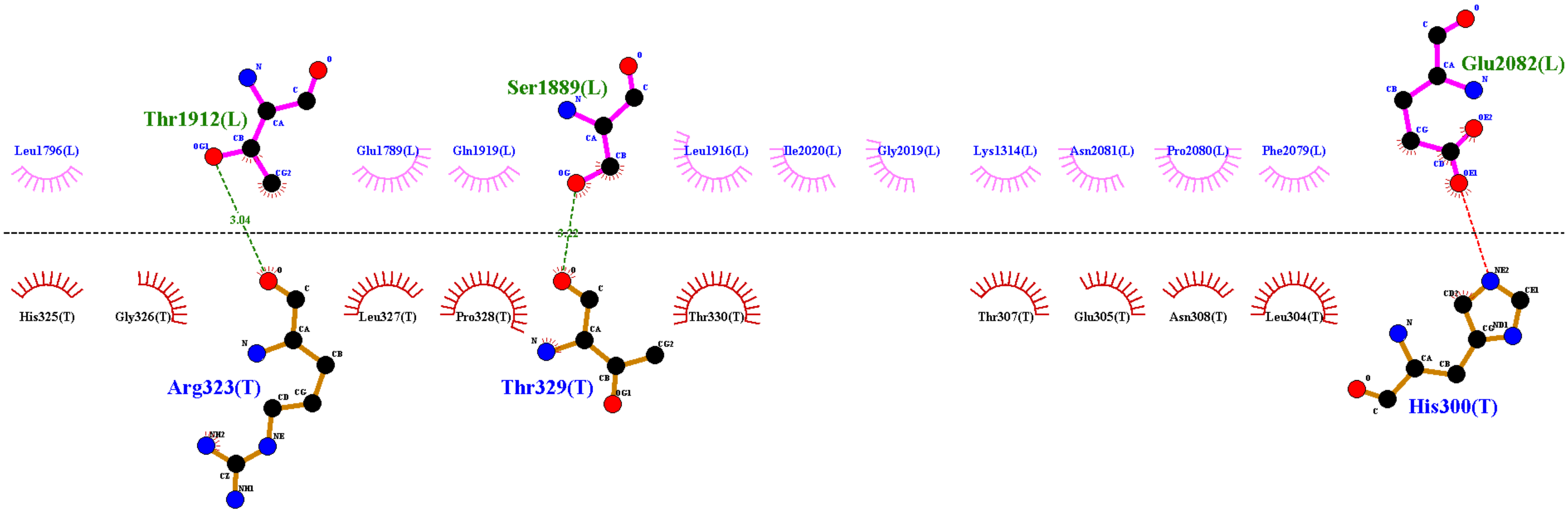

Supplementary Figure 5

Supplementary Figure 5. The T chain represents LRRK2, and the H chain represents TFEB. The stick structure indicates the interacting amino acid residues. In the red-black diagram, the red dashed lines represent salt bridge interactions, while the green dashed lines represent hydrogen bond interactions. For example, the negatively charged GLU2082 of LRRK2-wt forms a salt bridge interaction with the positively charged HIS300 of the TFEB protein. Additionally, SER1889 and THR1912 of LRRK2 form hydrogen bond interactions with ARG323 and THR329 of TFEB.

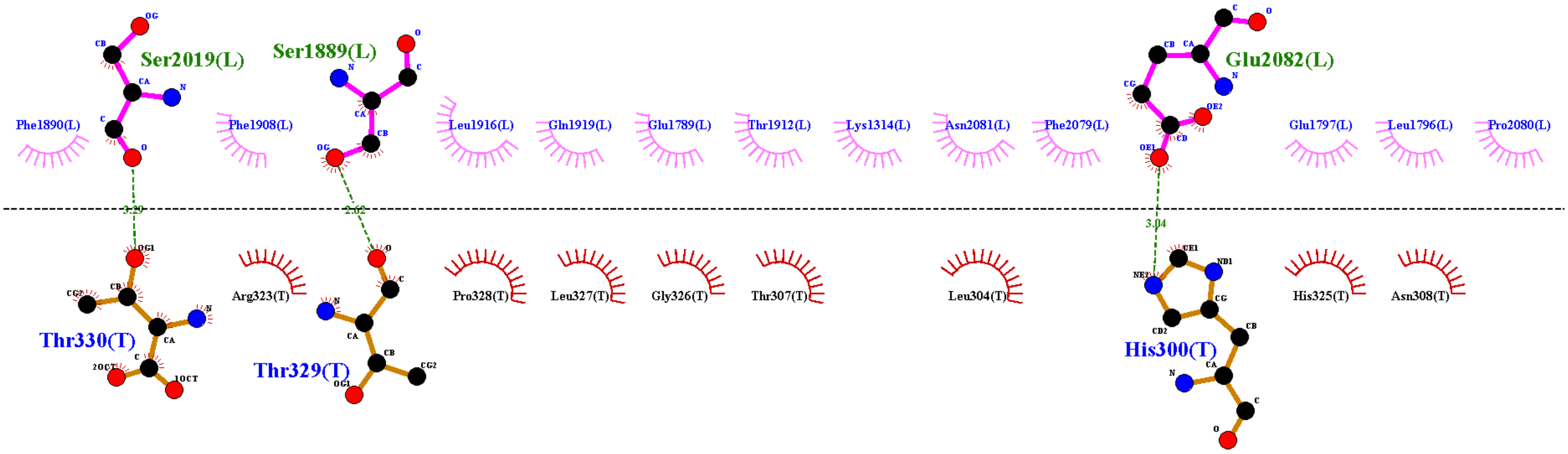

Supplementary Figure 6

Supplementary Figure 6. The T chain represents the LRRK2 G2019S mutation, and the H chain represents TFEB. The stick structure indicates the interacting amino acid residues. In the red-black diagram, the red dashed lines represent salt bridge interactions, and the green dashed lines represent hydrogen bond interactions. Specifically, the HIS300, THR329, and THR330 of the TFEB protein form hydrogen bond interactions with GLU2082, SER1889, and SER2019 of the LRRK2 G2019S mutant protein.

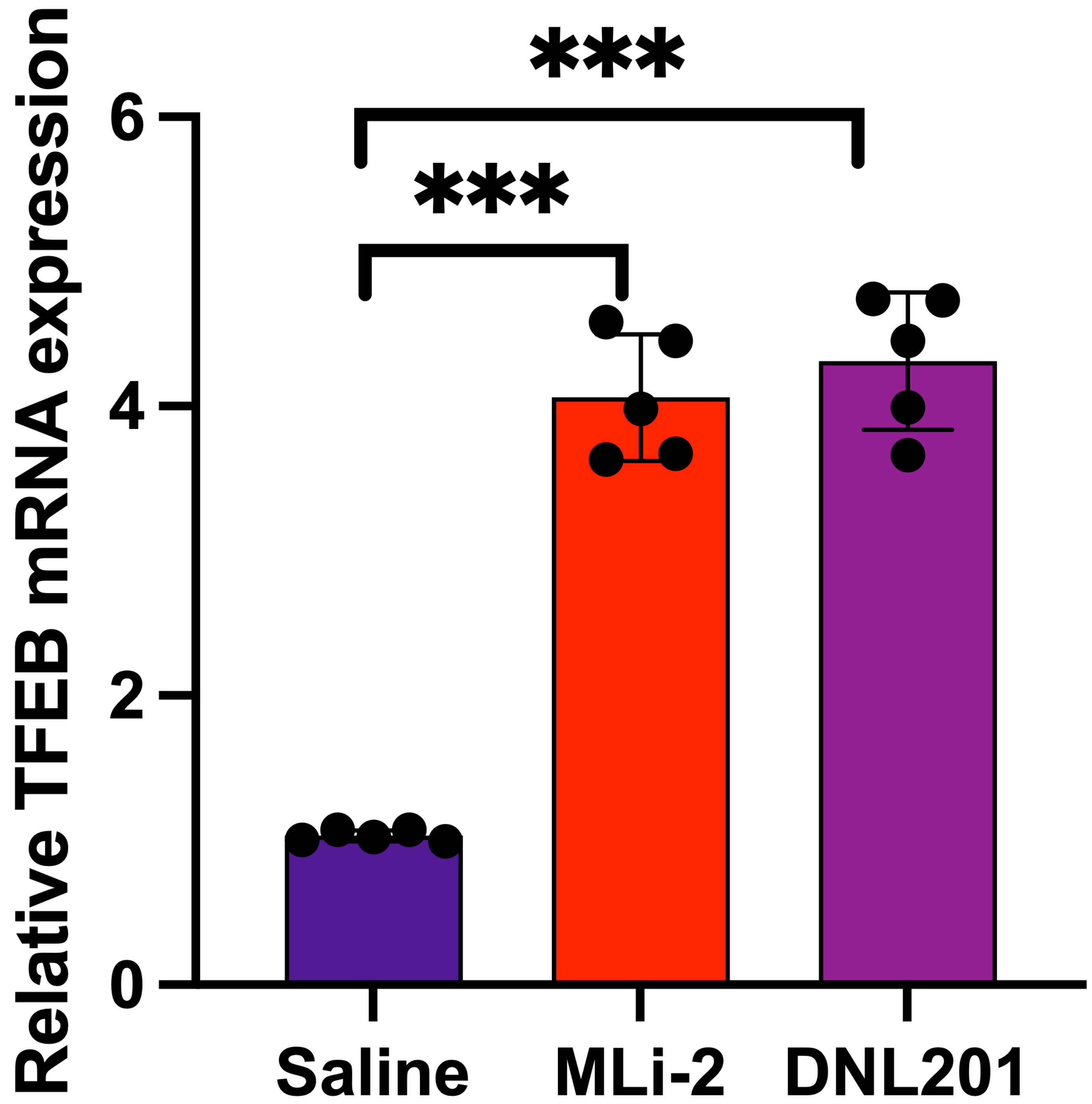

Supplementary Figure 7

Supplementary Figure 7. The relative mRNA level of TFEB in the hippocampus was confirmed by RT-qPCR. Data were presented as means ± SD. The experiments were carried out 5 times. The difference in folds is statistically significant. ****P* < 0.001.

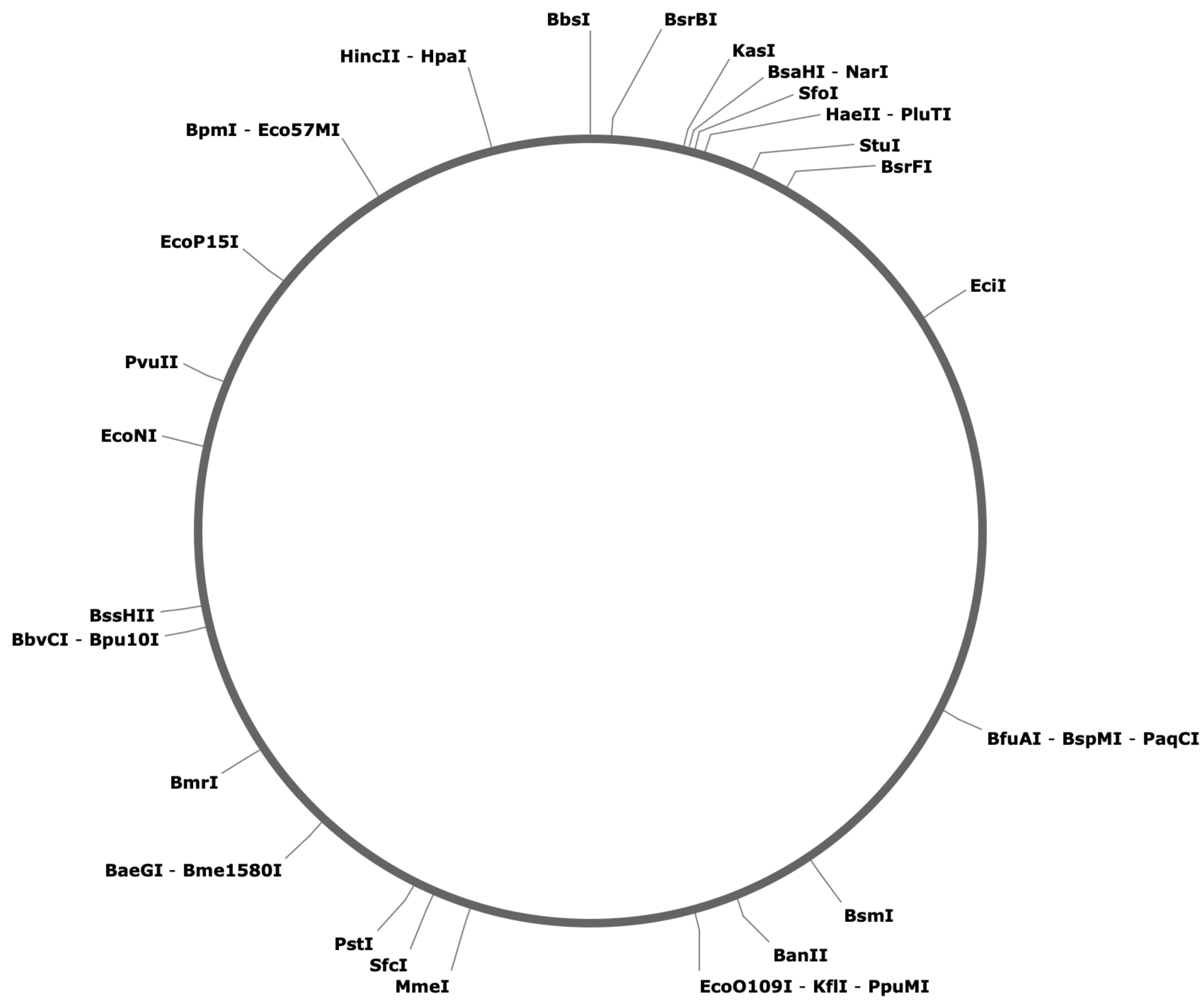

Supplementary Figure 8

Supplementary Figure 8. (A) Schematic representation of TFEB CRISPRa plasmids.

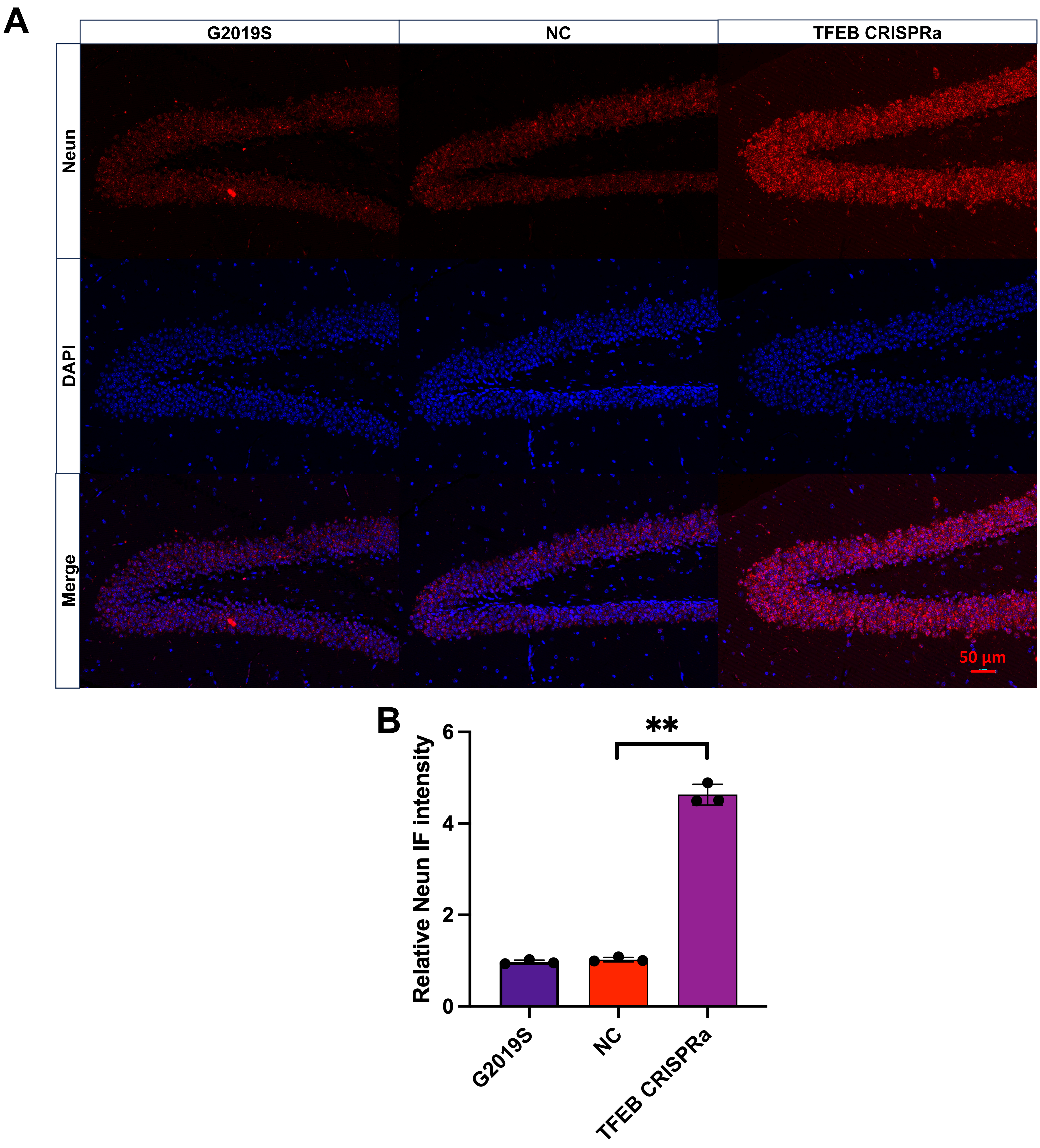

Supplementary Figure 9

Supplementary Figure 9. (A) Confocal immunofluorescence images revealed the expression level of Neun in the hippocampus after injections. Red: anti-Neun. Scale bar = 50 μm. (B) The relative fluorescence intensities of Neun were quantified in the hippocampus. Data were presented as means ± SD. The experiments were carried out 3 times. The difference in folds is statistically significant. $^{**}P < 0.01$.

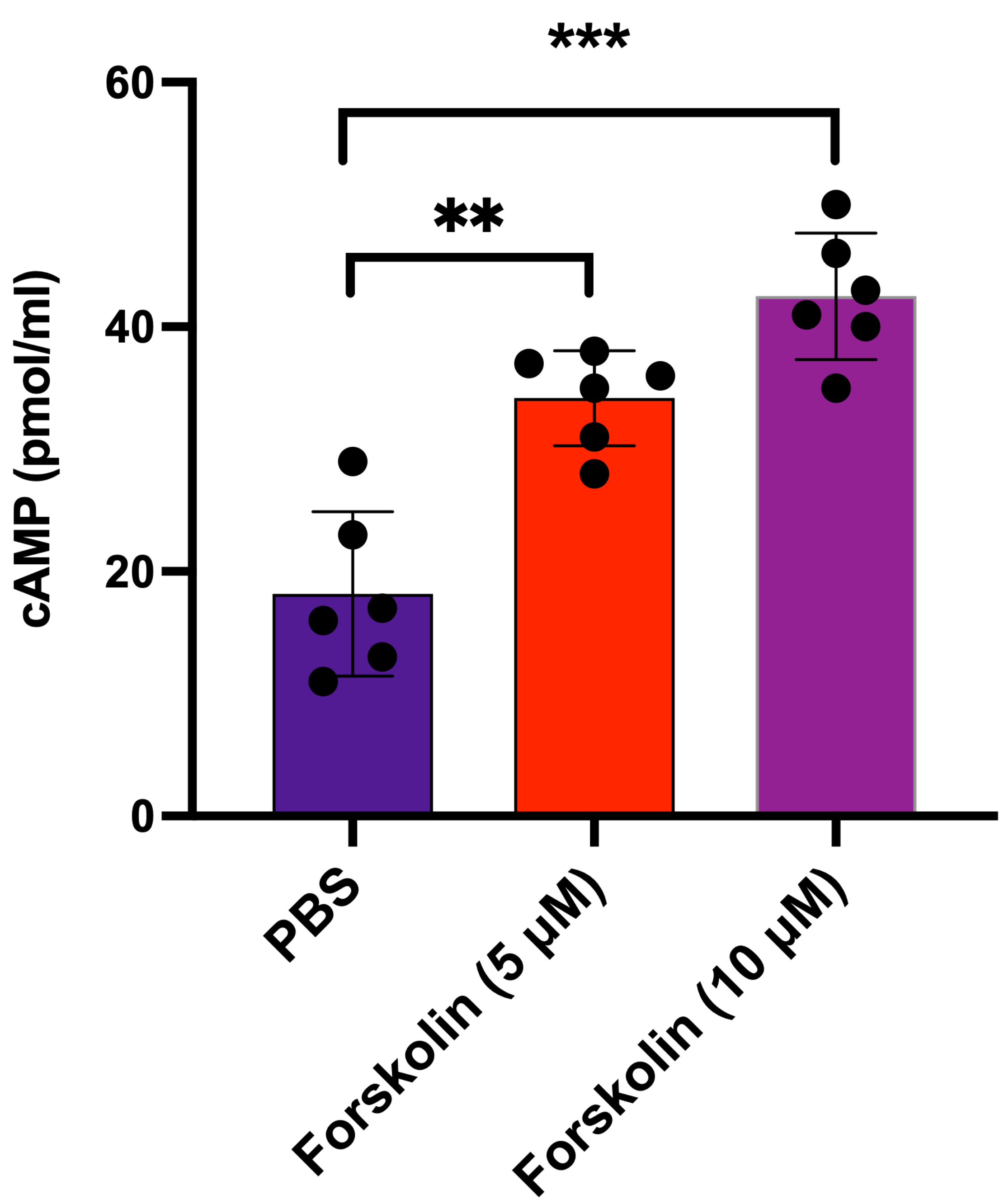

Supplementary Figure 10

Supplementary Figure 10. Cellular cAMP levels were quantified by ELISA (units: pmol/ml) under three conditions: PBS, forskolin 5 µM, and forskolin 10 µM. n = 6 biologically independent samples per group. Data were presented as means ± SD. Statistics: one-way ANOVA with Tukey's post hoc test; ** $P < 0.01$; *** $P < 0.001$.

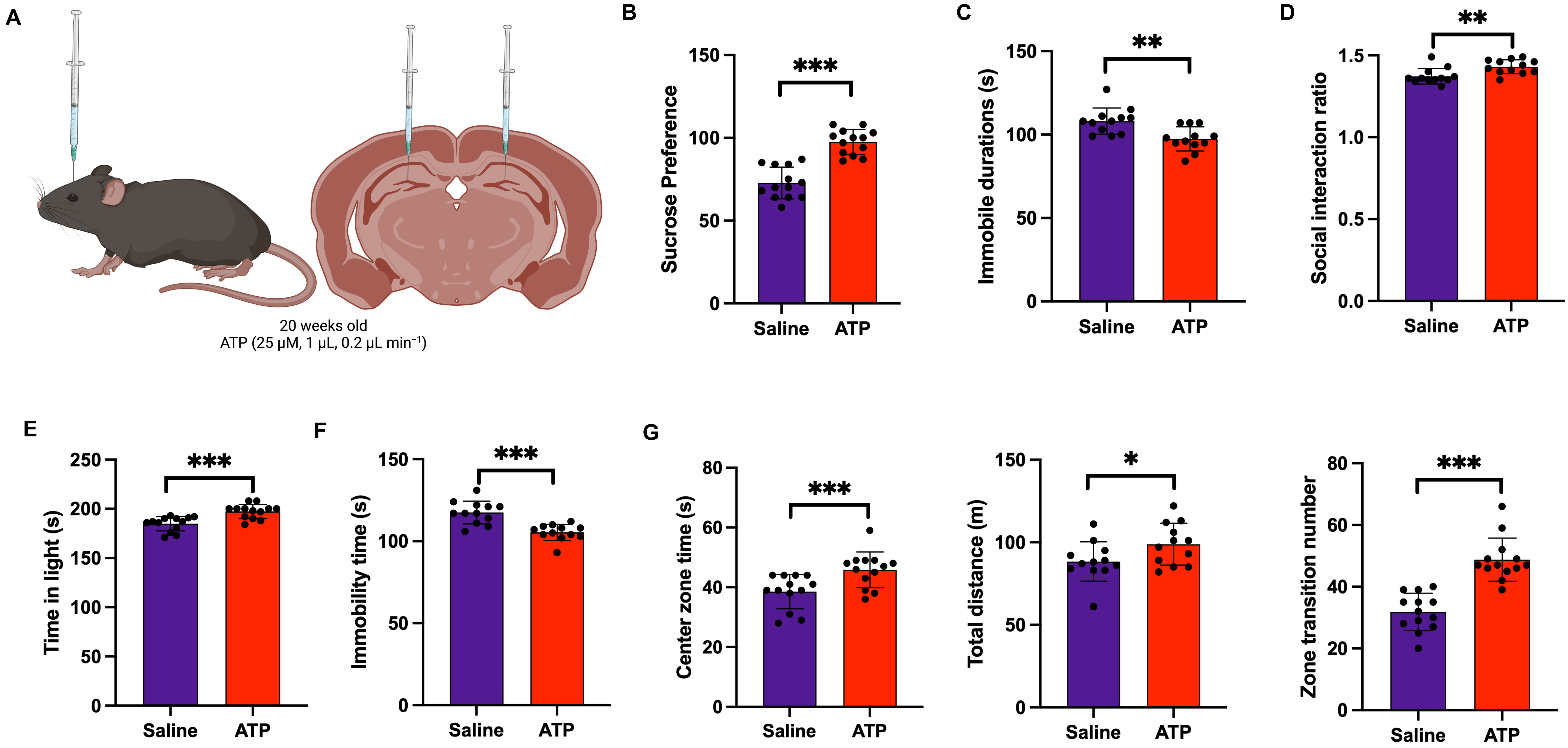

Supplementary Figure 11

Supplementary Figure 11. (A) Schematic of the hippocampal ATP intervention paradigm. (B–G) After hippocampal ATP treatment and TFEB CRISPRa injections, depression-related behaviors were assessed: SPT (B), FST (C), SIT (D), LDT (E), TST (F), and OFT (G). Data presentation: mean ± SD. Sample size: n = 12 mice per group. Statistics: one-way ANOVA with Tukey's post hoc test. Significance: * $P < 0.05$; ** $P < 0.01$; *** $P < 0.001$.

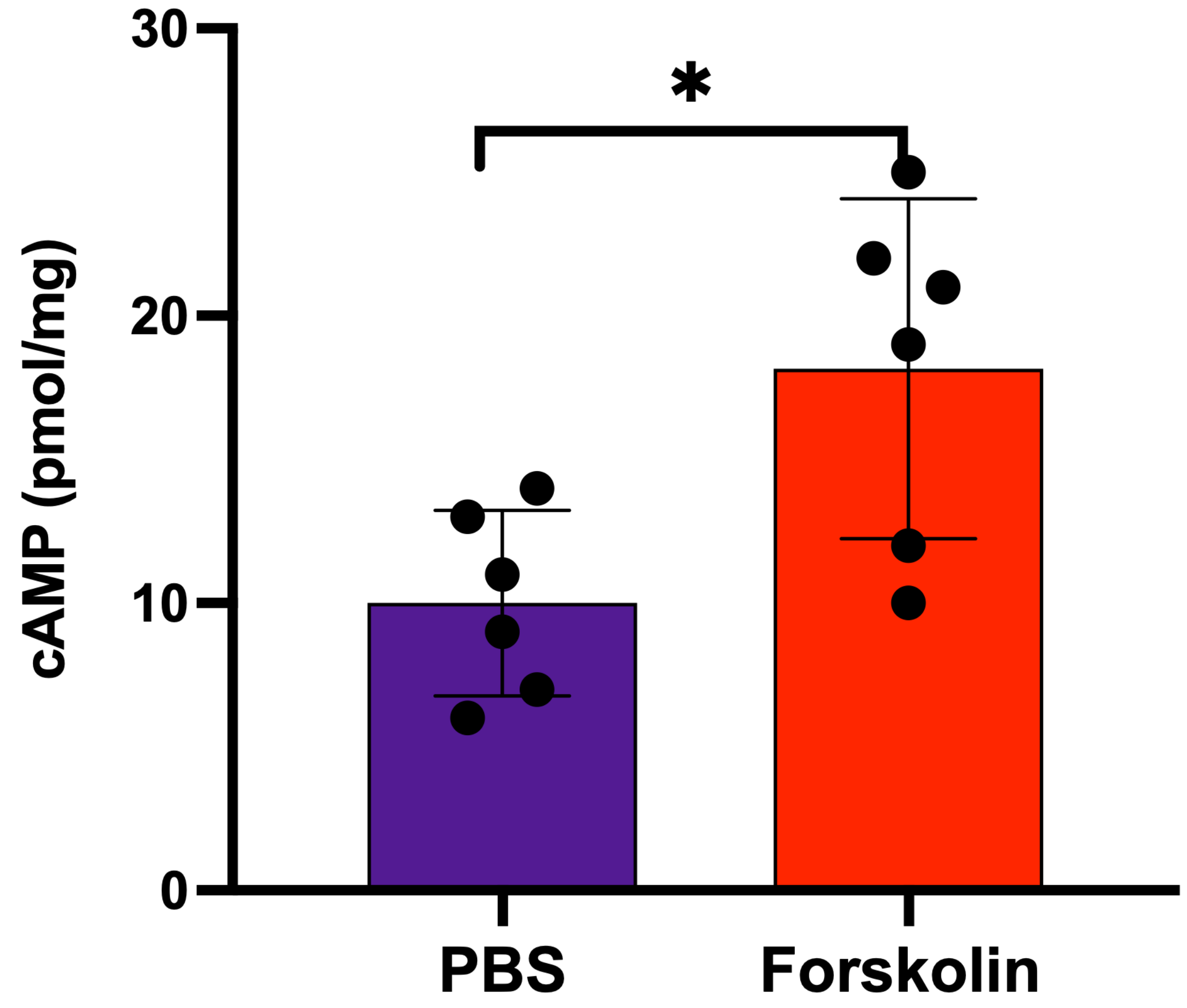

Supplementary Figure 12

Supplementary Figure 12. cAMP was quantified by ELISA (units: pmol/mg) in two conditions: PBS and forskolin injection in LRRK2 G2019S mice. n = 6 biologically independent samples per group. Data were presented as means ± SD. Statistics: unpaired two-tailed t-test; * $P < 0.05$.

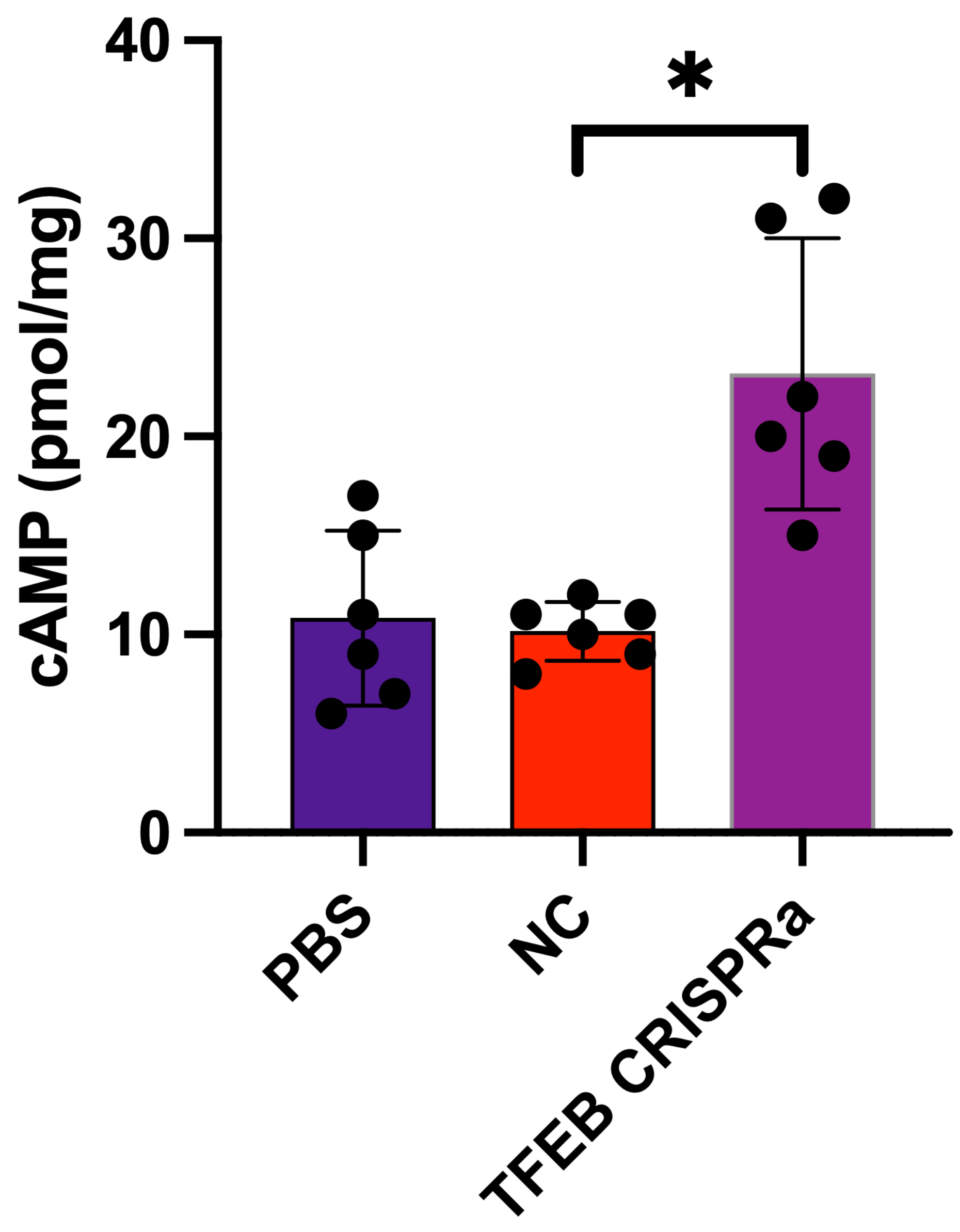

Supplementary Figure 13

Supplementary Figure 13. cAMP levels (pmol/mg) in LRRK2 G2019S mice following hippocampal injections of PBS, NC, or TFEB CRISPRa which was quantified by ELISA. Data are mean ± SD. n = 6 biologically independent mice per group. Statistics: one-way ANOVA with Tukey’s post hoc test; * $P < 0.05$.

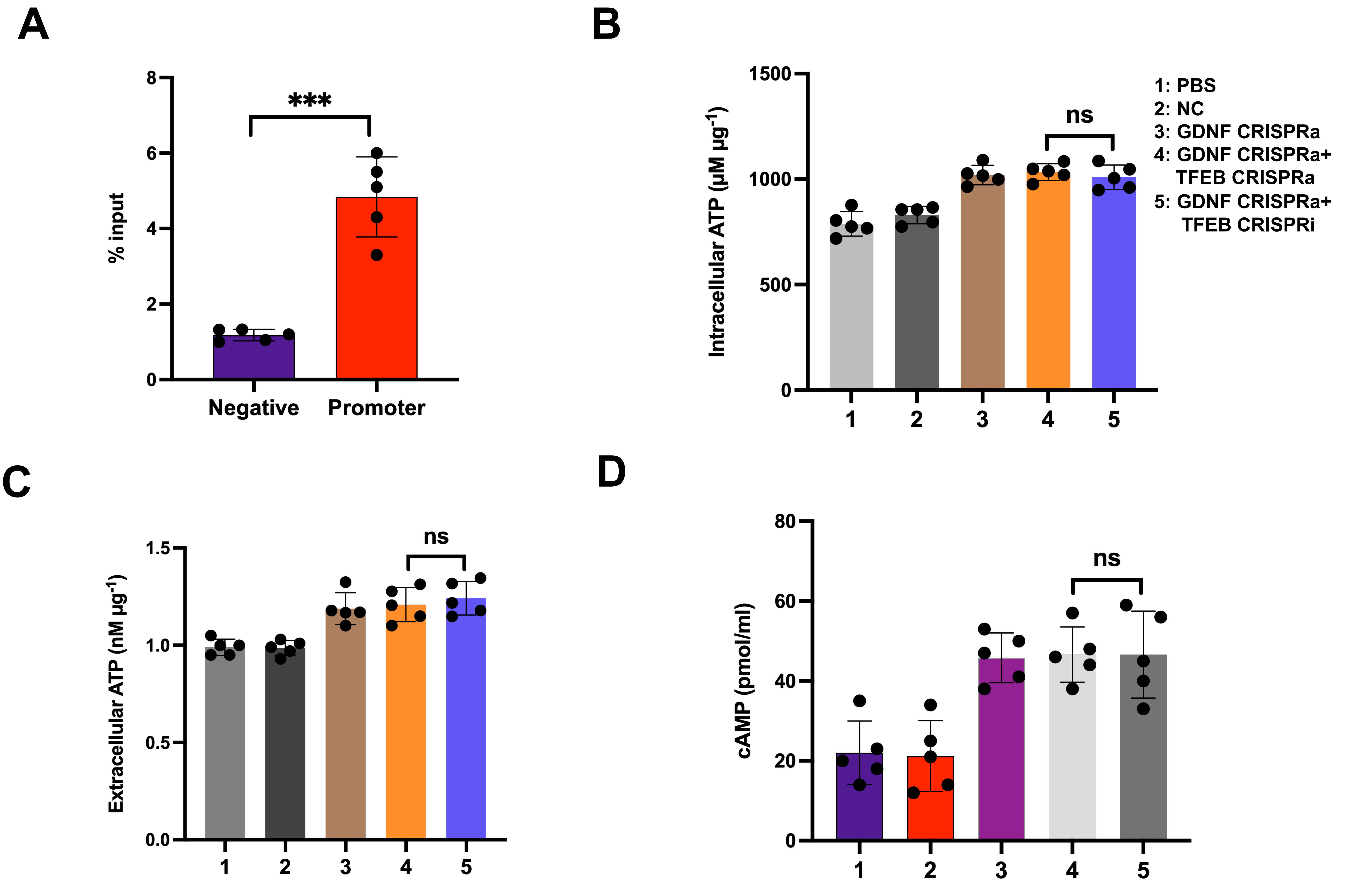

Supplementary Figure 14

Supplementary Figure 14. (A) ChIP–qPCR analysis showing enrichment of TFEB at the GDNF promoter region compared with a distal negative-control genomic region, presented as % input. Astrocytes were first transfected with GDNF CRISPRi for 48 h, then with TFEB CRISPRa or CRISPRi for an additional 48 h. (B-C) Intracellular and extracellular ATP in astrocytes and (D) cAMP measurements via ELISA. Data are mean ± SD. n = 5 biologically independent experiments per group. Statistics: one-way ANOVA with Tukey's post hoc test; *** $P < 0.001$. ns = not significant.

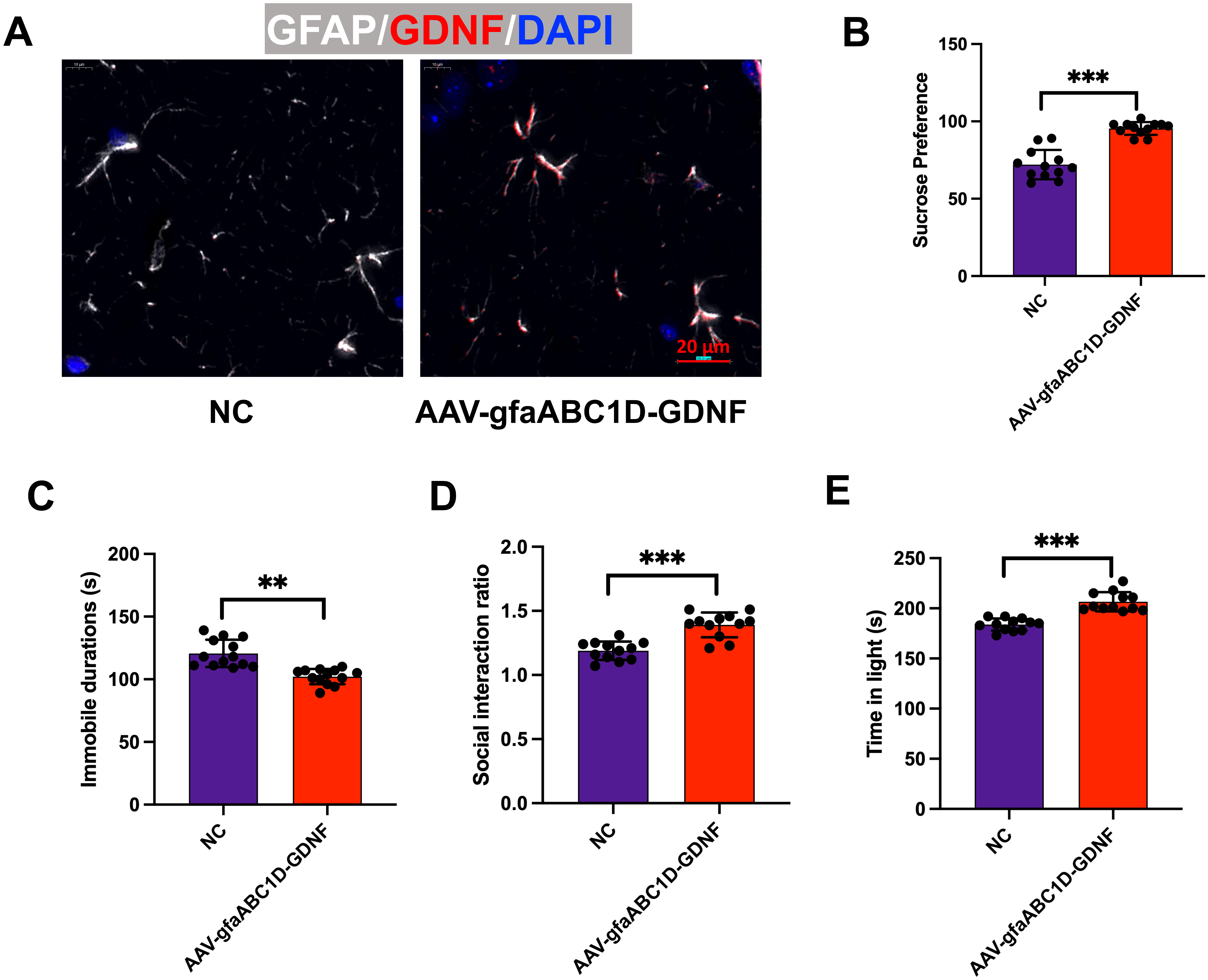

Supplementary Figure 15

Supplementary Figure 15. (A) Representative immunofluorescence images showing GFAP (gray/white), GDNF (red), and DAPI (blue) in the hippocampus of mice injected with NC or AAV-gfaABC1D-GDNF. (B–E) Behavioral readouts assessed 3 weeks after hippocampal injection: SPT (B), FST (C), SIT (D), and LDT (E). Data are presented as mean ± SD. Statistical significance was determined by two-tailed unpaired Student's t-test. $^{**}p < 0.01$, $^{***}p < 0.001$.

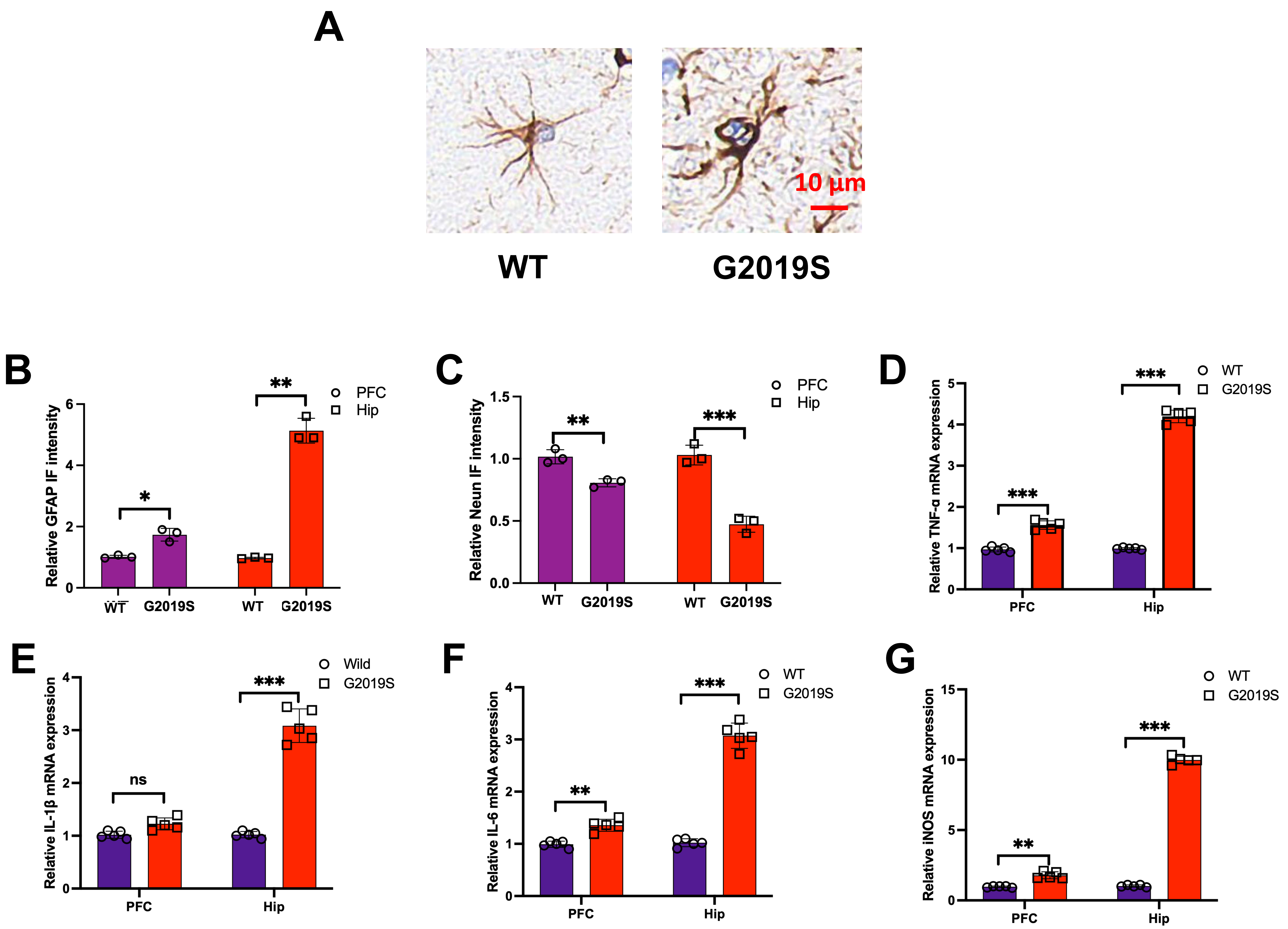

Supplementary Figure 16

Supplementary Figure 16. (A) Representative high-magnification images of GFAP-positive astrocytes in the hippocampus of WT and LRRK2 G2019S mice. Scale bar, 10 µm. (B–C) Quantification of GFAP (B) and NeuN (C) immunofluorescence signal intensity in PFC and Hip of WT and LRRK2 G2019S mice. Mean fluorescence intensity was measured within the indicated ROI after background subtraction and normalized to the WT group within each brain region. (D-G) RT-qPCR of inflammatory transcripts normalized to GAPDH: TNF-α (D), IL-1β (E), IL-6 (F), and iNOS (G). Data are mean ± SD. * $P < 0.05$, ** $P < 0.01$, *** $P < 0.001$. ns = not significant.

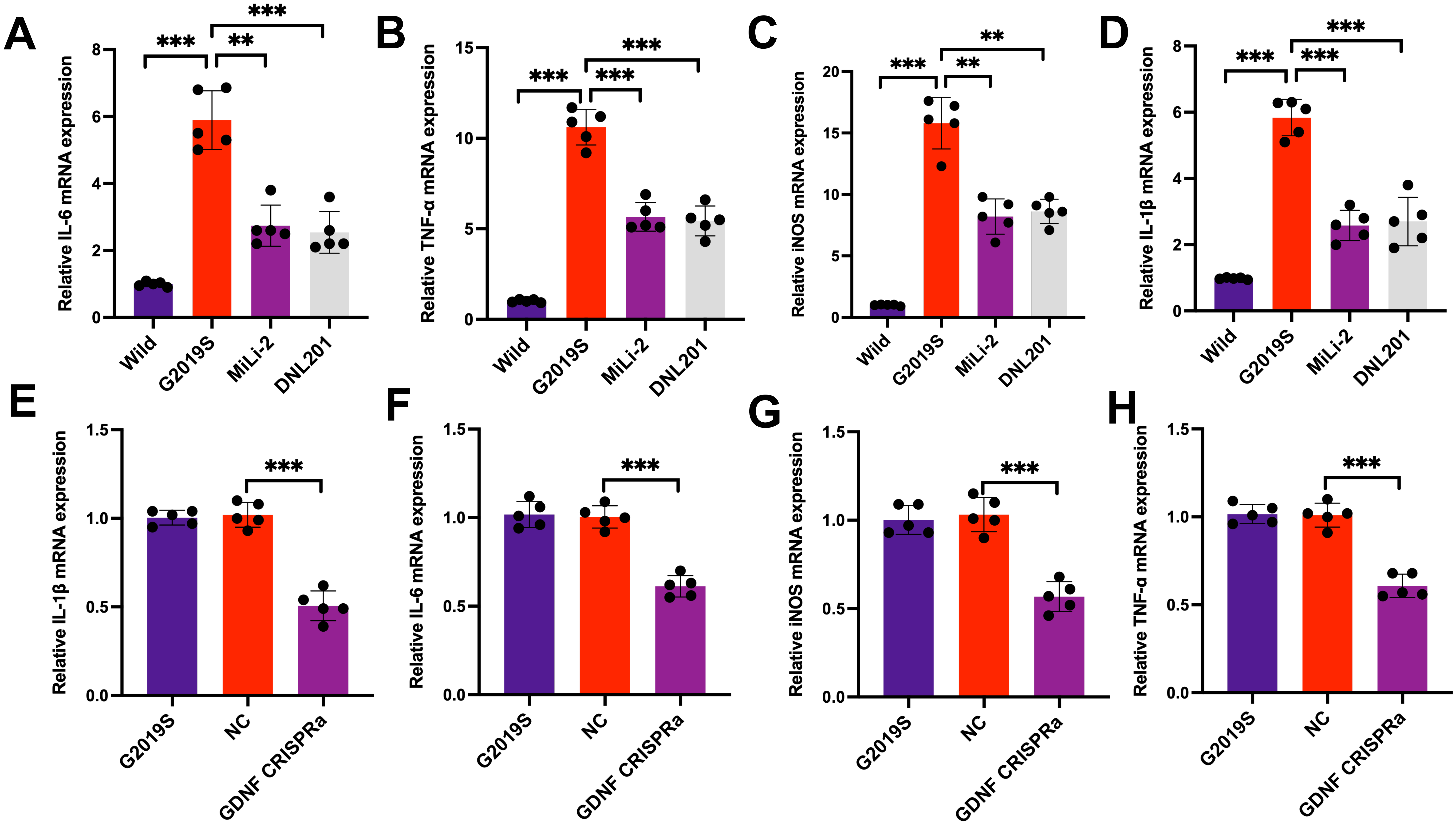

Supplementary Figure 17

Supplementary Figure 17. (A-E) RT-qPCR of inflammatory transcripts normalized to GAPDH after MLi-2 and DNL201 treatment: IL-6 (A), TNF-α (B), iNOS (C), IL-1β (D). (E-H) RT-qPCR of inflammatory transcripts normalized to GAPDH after GDNF CRISPRa transfection: IL-1β (E), IL-6 (F), iNOS (G), TNF-α (H). n=5. Data are mean ± SD. ** $P < 0.01$, *** $P < 0.001$.

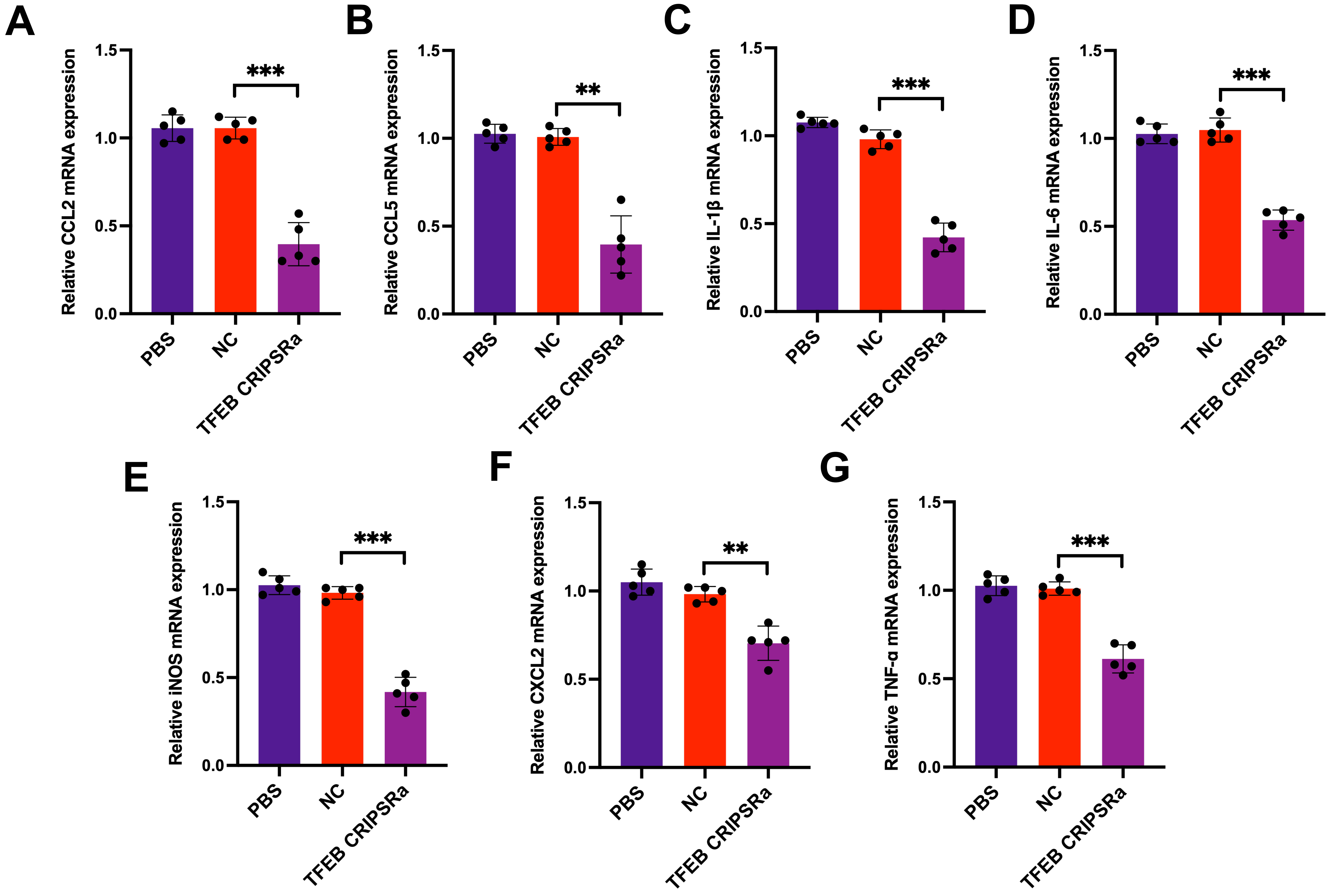

Supplementary Figure 18

Supplementary Figure 18. (A-G) RT-qPCR for inflammatory transcripts normalized to GAPDH: CCL2 (A), CCL5 (B), IL-1β (C), IL-6 (D), iNOS (E), CXCL2 (F), TNF-α (G). n=5. Data were presented as means ± SD. ** $P < 0.01$, *** $P < 0.001$.
